# *EMF2* deficiency disrupts epigenetic and chromatin organization landscapes, blocking root regeneration competence in Arabidopsis

**DOI:** 10.1111/nph.71298

**Published:** 2026-05-28

**Authors:** Zhidan Wang, May Avraham, Tali Mandel, Leor Eshed Williams, Chang Liu

**Affiliations:** ^1^ Department of Epigenetics, Institute of Biology University of Hohenheim Stuttgart 70599 Germany; ^2^ The Robert H. Smith Institute of Plant Sciences and Genetics in Agriculture The Hebrew University of Jerusalem Rehovot 76100 Israel; ^3^ Cluster of Excellence GreenRobust University of Hohenheim Stuttgart 70599 Germany

**Keywords:** Arabidopsis, BAC‐based Capture Hi‐C, EMF2, H3K27me3, root regeneration, three‐dimensional chromatin organization

## Abstract

- Plant *de novo* organogenesis depends on callus formation, yet the epigenetic mechanisms governing organ regeneration remain poorly understood. EMBRYONIC FLOWER 2 (EMF2), a core component of Polycomb Repressive Complex 2, mediates transcriptional repression through H3K27me3 and is essential for root regeneration in Arabidopsis. Here, we investigate how EMF2‐mediated H3K27me3 shapes chromatin architecture and gene expression during root regeneration.
- We combined time‐course transcriptome profiling, H3K27me3 chromatin immunoprecipitation, *in situ* Hi‐C, and a probe hybridization‐based Capture Hi‐C approach to analyze chromatin organization and gene expression dynamics in wild‐type and *emf2* calli during root induction.
- Although *emf2* calli lost root regeneration capacity, they retained responsiveness to root induction signals. Despite large‐scale reduction of H3K27me3 across the *emf2* genome, many genes remained transcriptionally inactive, coinciding with the formation of new long‐range chromatin interactions and weakened intra‐ and peri‐domain contacts. Genes with low basal transcription were preferentially derepressed following extensive H3K27me3 loss.
- Our results demonstrate that large‐scale reduction of H3K27me3 in *emf2* drives dynamic reorganization of chromatin architecture in Arabidopsis callus, providing new insights into how histone modification and three‐dimensional chromatin topology coordinately regulate gene expression during plant regeneration.

## Graphical abstract

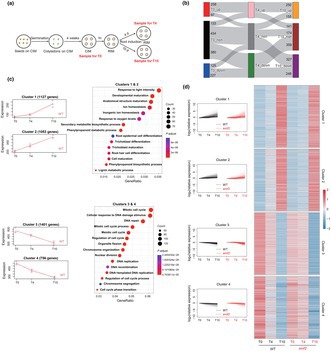

## Introduction

Plants possess high pluripotency, enabling them to regenerate and thereby overcome their limitations as sessile organisms (Ikeuchi *et al*., 2019). *De novo* root regeneration (DNRR) is the process by which adventitious roots develop in response to tissue damage, supplementing the plant's root system. These roots can regenerate either directly from detached or wounded plant tissues, or indirectly from an intermediate mass of proliferating cells termed callus (Klerk *et al*., 1999; Sugimoto *et al*., 2010; Xu & Huang, 2014; Asghar *et al*., 2025). The capacity of DNRR is employed in agriculture to clonally propagate several important crops and trees, and indirect DNRR is widely used to generate stable transgenic or gene‐edited plants through *in vitro* tissue culture (Chen *et al*., 2014; Jing *et al*., 2020).

In indirect DNRR, explants such as cut leaves or cotyledons are first cultured on callus‐inducing medium (CIM), which contains the phytohormones auxin and cytokinin, resulting in the formation of a mass of pluripotent cells called callus at the wound sites. Following culturing on a root‐inducing medium (RIM) with low auxin levels, the callus regenerates roots (Skoog & Miller, 1957; Yu *et al*., 2017). In Arabidopsis, indirect DNRR involves two cell fate transition steps (Sugimoto *et al*., 2010; Yu *et al*., 2017; Zhai & Xu, 2021; Liu *et al*., 2023). In the first step, auxin activates *WUSCHEL RELATED HOMEOBOX 11* (*WOX11*) and *WOX12*, leading to the transition of competent cells into founder cells (Atta *et al*., 2009; Liu *et al*., 2014; Xu, 2018). Then, the expression of *WOX5*, *WOX7*, *LATERAL ORGAN BOUNDARIES‐DOMAIN 16* (*LBD16*), and *LBD29* increases, which promotes founder cell proliferation and callus formation (Fan *et al*., 2012; Hu & Xu, 2016; Liu *et al*., 2018, 2023). On RIM, callus undergoes cell division and gives rise to root apical meristem, during which *LBD16* expression decreases, and the expression of *WOX5* and *WOX7* is gradually restricted to the stem cell niche (Hu & Xu, 2016; Xu, 2018; Jing *et al*., 2020; Liu *et al*., 2023). In addition, root stem cell regulators, such as *PLETHORA 1* (*PLT1*), *PLT2*, *SCARECROW* (*SCR*), and *SHORT ROOT* (*SHR*), are activated to establish competency to regenerate (Della Rovere *et al*., 2015; Kareem *et al*., 2015; Shimotohno *et al*., 2018). Although the molecular mechanisms underlying callus formation have been extensively studied, how roots are regenerated from callus remains largely unclear.

Epigenetic landscapes accompany cell fate transition, and histone modification dynamics play a crucial role in gene regulation during plant regeneration (Lee & Seo, 2018; Liu *et al*., 2023; Chen *et al*., 2024). In Arabidopsis, the histone methyltransferase ARABIDOPSIS TRITHORAX‐RELATED 2 (ATXR2) enables the activation of *LBD16* and *LBD29* by the deposition of H3K36me3 at their promoters (Lee *et al*., 2017). Furthermore, JUMONJI C DOMAIN‐CONTAINING PROTEIN 30 (JMJ30), a histone lysine demethylase, functions in promoting the expression of *LBDs* through the removal of methyl groups from H3K9me3 (Lee *et al*., 2018). The Arabidopsis histone acetyltransferase, HISTONE ACETYLTRANSFERASE OF THE GNAT/MYST SUPERFAMILY 1 (HAG1)/GENERAL CONTROL NONREPRESSED 5 (AtGCN5), is required for pluripotency acquisition during callus formation by catalyzing histone acetylation at several root‐meristem gene loci including *WOX5*, *WOX14*, *SCR*, *PLT1*, and *PLT2* (Kim *et al*., 2018). ARABIDOPSIS TRITHORAX 4 (ATX4) catalyzes the trimethylation of H3K4 and is primarily associated with gene activation (Foroozani *et al*., 2021). The downregulation of *ATX4* on CIM results in the reduction of H3K4me3 and expression levels of leaf identity genes, enabling the formation of callus from leaf explants (Lee *et al*., 2019).

Polycomb Repressive Complex 2 (PRC2), responsible for catalyzing the repressive modification H3K27me3 and associated with three‐dimensional chromatin organization, has been implicated in callus formation in Arabidopsis, and genome‐wide reprogramming of H3K27me3 is critical for cell fate transitions in this process (He *et al*., 2012; Kim *et al*., 2012; Liu *et al*., 2016; Huang *et al*., 2021; Uckelmann & Davidovich, 2021). During callus formation, certain auxin pathway genes and many root‐regulatory genes are derepressed through H3K27 demethylation; conversely, a number of leaf identity genes are suppressed by PRC2‐mediated H3K27me3 for the acquisition of pluripotency (He *et al*., 2012). The PRC2 mutants *curly leaf swinger* (*clf swn*) and *embryonic flower 2* (*emf2*) show defects in callus formation from leaf blades and/or cotyledons, likely due to the failure to repress leaf identity gene expression (He *et al*., 2012). Despite the established role of PRC2‐mediated H3K27me3 in callus formation, their involvement in organ regeneration remains largely unexplored. In our previous work, we successfully generated callus from *emf2‐1* by directly germinating seeds on CIM to promote the proliferation of embryonic cells, followed by excision of immature cotyledons and re‐culturing them on CIM. Interestingly, when these *emf2* calli were transferred to RIM, they failed to regenerate root, indicating an important role for EMF2‐PRC2 in the acquisition of new cell identity during root induction (Mandel *et al*., 2025). Here, by characterizing a time‐course transcriptomic analysis, along with H3K27me3 profiling and *in situ* Hi‐C analysis during root induction, we elucidated how H3K27me3‐mediated chromatin organization functions in gene regulation during plant regeneration.

## Materials and Methods

### Plant materials and growth conditions

The Columbia‐0 (Col‐0) ecotype of *Arabidopsis thaliana* (L.) Heynh. was used in this study. A published mutant of the *EMF2* gene (*AT5G51230*), *emf2‐1* (CS16238) was ordered from Arabidopsis Biological Resource Center (ABRC) (Yang *et al*., 1995). The mutant was genotyped according to the reference (Calonje *et al*., 2008), and the primer sequences were 5′‐AACAACAAATTGCAGAAGACTGAAG‐3′ and 5′‐CTTGGATATCATTGTCTCAGTCTTG‐3′. Seeds were surface sterilized for 10 min in 3% sodium hypochlorite containing 0.1% Triton X‐100. After four times washing with double‐distilled water, they were used for tissue culture.

### Tissue culture

All tissue culture experiments were done in a growth room at 23°C under continuous light or dark conditions. Callus induction was performed as described (Mandel *et al*., 2025). Sterilized seeds from Col‐0 and *emf2‐1* were sown directly on CIM (Gamborg B5 medium with 0.5 g l^−1^ MES and supplemented with 2% dextrose, 0.3% phytagel, supplemented with 2.2 μM 2,4‐dichlorophenoxyacetic acid (2,4‐D) and 0.46 μM kinetin). Following 2 d of incubation at 4°C in the dark, plates were transferred to a growth room with continuous light. After 7 d, cotyledons with callus‐like formation were trimmed and transferred to a new CIM plate placed in dark. Then calli were re‐cultured to new CIM plates every 7–10 d, and 4‐wk old calli on CIM were harvested as samples for T0. Subsequently, the calli were transferred to RIM (Gamborg B5 medium supplemented with 0.5 μM NAA) and incubated under dark conditions for root induction. Calli grown on RIM for 4 and 10 d were harvested as samples for T4 and T10, respectively.

### RNA sequencing

For RNA extraction, 6–8 calli (*c.* 0.5 g fresh weight) were pooled to constitute one biological replicate. Samples were snap‐frozen in liquid nitrogen and ground into a fine powder. 100 mg of tissue powder was used for total RNA extraction with the RNeasy Plant Mini Kit (Qiagen) following the manufacturer's instructions. Contaminating genomic DNA was removed from the RNA samples by treatment with RNase‐free DNase (Qiagen). Each RNA sample was loaded on agarose gel for integrity validation and quantified using NanoDrop.

RNA sequencing (RNA‐seq) was performed with three biological replicates per sample at Macrogen Europe B.V. (the Netherlands) according to Illumina's protocols. TruSeq RNA V2 sample prep kit (RS‐122) was used. Total RNA was polyA‐selected, followed by fragmentation and random hexamer primed reverse transcription. After second‐strand synthesis, indexed adapters were added, cDNA was amplified by PCR and samples were loaded onto the Illumina's NovaSeq system.

Reads were aligned against the Araport11 annotation using tophat2 (v.2.1.1) with default parameters, and were subsequently assigned to genes with the R package GenomicAlignments (Kim *et al*., 2013; Lawrence *et al*., 2013; Cheng *et al*., 2017). Differentially expressed genes (DEGs) were further identified using the R package deseq2 (Love *et al*., 2014), with the criteria of log_2_ (Fold Change) (log_2_ FC) > 1.6 and *q* < 0.01 to obtain upregulated and downregulated genes. For time‐course gene expression analysis, significant genes were identified using the R package maSigPro, with the following parameters: MT.adjust = ‘BH’, *Q* = 0.01, min.obs = 6, and further categorized into four clusters with cluster.method = ‘hclust’ (Conesa *et al*., 2006).

### Protein extraction and western blot

Histones were enriched according to a previously published method (Zhao & Grafi, 2000), with some modifications. 2 g of frozen calli (corresponding to *c.* 25–40 calli) were homogenized and transferred to 20 ml of NETN buffer (100 mM NaCl, 1 mM EDTA, 20 mM Tris–HCl, pH 8.0, 0.5% NP‐40, 200 mM PMSF, and protease inhibitor) containing 2.6% trichloroacetic acid (TCA) and incubated overnight at 4°C with constant shaking. The samples were filtered through miracloth (Millipore), centrifuged at 13 000 ***g*** for 10 min, and the supernatants were collected. To precipitate acid‐soluble proteins, TCA was slowly added to the supernatants to a final concentration of 25%, followed by incubation at 4°C for 1 h with constant shaking. Proteins were then precipitated by centrifugation at 13 000 ***g*** for 10 min, washed with 5 ml acetone, and incubated overnight at −20°C. The following day, the pellet was washed twice more with 1 ml acetone, air‐dried, and dissolved in 250–500 μl Laemmli buffer (65 mM Tris–HCl, pH 6.8, 10% glycerol, and 2% SDS). Protein quantification was performed using a BCA kit (Thermo Fisher Scientific, Rockford, IL, USA) and a NanoDrop spectrophotometer.

For histone western blot, histone samples were subjected to 18% acrylamide SDS‐PAGE and subsequently transferred to a nitrocellulose membrane (BA‐S 85; Whatman/Cytiva, Marlborough, MA, USA). The blots were blocked with 4% non‐fat dry milk and incubated with the primary antibody for 1 h. After washing, the membrane was incubated with the secondary antibody for 30 min. The following antibodies were used to detect histone H3 and different modifications: for H3 Abcam (Cambridge, UK), ab1791 (1 : 10 000); for H3K27me3 Millipore, 07‐499 (1 : 1000); for H3K4me3 Abcam, ab8580 (1 : 5000). Following an additional wash, the blots were visualized using chemiluminescence detection (Biological Industries). Uncropped western blot images were included in Fig. S12.

For CRWN1 detection, total protein was extracted from 10‐d‐old Arabidopsis shoots by homogenization in extraction buffer (50 mM Tris–HCl, pH 7.5, 150 mM NaCl, 0.1% TWEEN 20, 0.1% 2‐mercaptoethanol, 0.1 mM PMSF and 1× protease inhibitor cocktail (Roche)). After centrifugation, the supernatant was collected for western blot analysis. Anti‐CRWN1 antibodies were generated against a synthesized peptide MSTPLKVWQRWSTPT, following published research (Blunt *et al*., 2020). The peptide was injected into rabbits, and the resulting antisera were affinity‐purified and validated (BioGenes, Berlin, Germany). The CRWN1 antibody was used at a dilution of 1 : 2000. A HRP‐conjugated goat anti‐rabbit secondary antibody (A6154; Sigma‐Aldrich) was applied at a 1 : 5000 dilution. Signal detection was performed via chemiluminescence, followed by Coomassie Blue staining of the membrane as a loading control.

### Chromatin immunoprecipitation and library sequencing

Calli and 10‐d‐old Arabidopsis shoots were harvested and fixed in 35 ml MC buffer (10 mM sodium phosphate, pH 7.0, 50 mM NaCl and 100 mM sucrose) containing 1% formaldehyde for 30 min under vacuum. Fixation was terminated by replacing the solution with 0.15 M glycine in MC buffer and vacuum for 10 min. After washing, samples were dried on KimWipe paper, and then snap‐frozen in liquid nitrogen. Subsequently, fixed samples were homogenized in liquid nitrogen using a pre‐chilled mortar and pestle and resuspended in nuclei isolation buffer (20 mM HEPES, pH 8.0, 250 mM sucrose, 1 mM MgCl_2_, 5 mM KCl, 40% glycerol, 0.25% Triton X‐100, 0.1 mM PMSF, 0.1% 2‐mercaptoethanol) and filtered with double‐layered miracloth (Millipore). Nuclei were then resuspended in 0.5 ml sonication buffer (10 mM potassium phosphate, pH 7.0, 0.1 mM NaCl, 0.5% sarkosyl, 10 mM EDTA), and chromatin was sheared by using a QSONICA sonicator (Q800R3) to achieve average fragment size *c.* 400 base pairs (bp). After adding 50 μl of 10% Triton X‐100, the sheared chromatin solution was mixed with an equal volume of immunoprecipitation (IP) buffer (50 mM HEPES, pH 7.5, 150 mM NaCl, 5 mM MgCl_2_, 10 μM ZnSO4, 1% Triton X‐100, 0.05% SDS). For H3K27me3 immunoprecipitation, Pierce™ protein A/G magnetic beads (Thermo Fisher Scientific, Rockford, IL, USA), and anti‐H3K27me3 antibody (07‐449; Merck, Darmstadt, Germany) were added and incubated at 4°C for 2 h. In parallel, samples incubated with anti‐H3 antibody (ab1791; Abcam) were used as controls. For CRWN1, an endogenous antibody with confirmed specificity via western blot was used, which was generated following a published protocol (Blunt *et al*., 2020) and validated as described in the western blot in the ‘Materials and Methods’ section. After immunoprecipitation, the beads were washed at 4°C as follows: two times with IP buffer, one time with high salt buffer (IP buffer containing 500 mM NaCl) and one time with LiCl buffer (0.25 M LiCl, 1% NP‐40, 1% deoxycholate, 1 mM EDTA, 10 mM Tris–HCl pH 8.0) for 3 min each. Following a brief wash with TE buffer (10 mM Tris–HCl, pH 8.0, 1 mM EDTA), the beads were resuspended in 200 μl elution buffer (50 mM Tris–HCl, pH 8.0, 200 mM NaCl, 1% SDS, 10 mM EDTA) and incubated at 65°C for 6 h. After proteinase K treatment at 45°C for 1 h, DNA was purified with a MinElute PCR purification kit (Qiagen), and subsequently converted into sequencing libraries following the manual of NEBNext Ultra II DNA Library Prep Kit (NEB). The libraries were sequenced on an Illumina Novaseq instrument with 2 × 150 bp reads.

ChIP‐seq reads were mapped to the TAIR10 genome using bowtie 2 (v.2.2.4) (Langmead & Salzberg, 2012), and peak calling was done with macs2 v.2.1.1 (Zhang *et al*., 2008). To compare H3K27me3 enrichment between samples, the reads from anti‐H3K27me3 immunoprecipitation were normalized to the corresponding reads from anti‐H3 immunoprecipitation, and the differentially enriched sites were identified using the R package diffbind with default parameters (Rory Stark, 2017). The visualization of the read coverage over the genome was conducted by deepTools (Ramírez *et al*., 2016).

### Fluorescent *in situ* hybridization (FISH)

Bacterial artificial chromosome (BAC) probes for FISH were generated by nick translation according to the manufacturer's instructions (11 745 808 910; Roche). Green and red probes were labeled with digoxigenin‐11‐dUTP and dinitrophenol‐11‐dUTP, respectively. Labeled probes were pooled in a hybridization buffer (50% formamide, 10% dextran sulfate, 2× SSC, 125 μg ml^−1^ salmon sperm DNA, 0.125% SDS) to achieve a working concentration of 1 ng μl^−1^ for each BAC. The BACs used in this study are listed in Table S1.

For dual‐color FISH, fixed calli were chopped in GPB buffer (0.5 mM spermine tetrahydrochloride, 30 mM sodium citrate, 20 mM MOPS, 80 mM KCl, 20 mM NaCl, 0.5% Triton X‐100, pH 7.0) and filtered through a 40‐μm cell strainer. Nuclei were further purified by staining with DAPI (4′,6‐diamidino‐2‐phenylindole) and sorting with a cell sorter (Bio‐Rad S3e), and subsequently resuspended in 10 μl PBS. After incubation at 65°C for 30 min, the nuclei were mixed with 10 μl of 0.1 mg ml^−1^ RNase A and transferred onto a Superfrost Ultra Plus Adhesion Slide (Thermo Fisher Scientific, Braunschweig, Germany). Following 1 h incubation at 37°C in a hybridizer (model 07J91‐020; ThermoBrite, Richmond, IL, USA), the slide was washed with PBS and then dehydrated with a graded ethanol series. The subsequent hybridization and post‐hybridization wash steps were performed as previously described (Hu *et al*., 2019). Digoxigenin‐11‐dUTP‐labeled probes were detected using a mouse anti‐digoxin antibody (SAB4200669; Sigma), followed by a goat anti‐mouse Alexa Fluor 488‐conjugated antibody (A11017; Invitrogen). Dinitrophenol‐11‐dUTP‐labeled probes were detected with a rabbit anti‐dinitrophenyl antibody (A6430; Invitrogen), followed by a goat anti‐rabbit Alexa Fluor 546‐conjugated antibody (A11071; Invitrogen). All antibodies were diluted 1 : 500. Confocal images were acquired using a Zeiss LSM700 system, and image processing was performed with fiji image j.

### *In situ* Hi‐C and data analysis

*In situ* Hi‐C libraries were prepared as previously described with two replicates for each sample (Wang *et al*., 2023). Nuclei were isolated from 1 g fixed calli (corresponding to 15–20 calli) and resuspended with 150 μl 0.5% SDS and further split into three tubes. Followed by nuclei permeabilization at 62°C for 5 min, 145 μl water and 25 μl 10% Triton X‐100 were added to each tube, and incubated at 37°C for 15 min. Chromatin was then digested at 37°C with 50 U DpnII (NEB) overnight, after which the enzyme was inactivated by incubating at 62°C for 20 min. Subsequently, sticky ends filling and chromatin labeling were performed by adding 1 μl of 10 mM dTTP, 1 μl of 10 mM dATP, 1 μl of 10 mM dGTP, 10 μl of 1 mM biotin‐14‐dCTP, 29 μl water and 40 U Klenow fragment (Thermo Fisher), and incubating at 37°C for 2 h. For proximity ligation, 663 μl water, 120 μl 10× blunt‐end ligation buffer (300 mM Tris–HCl, 100 mM MgCl_2_, 100 mM DTT, 1 mM ATP, pH 7.8) and 40 U T4 DNA ligase (Thermo Fisher) were added, and incubated at room temperature for 4 h. After centrifugation, three tubes of nuclei pellet were resuspended and combined with 650 μl SDS buffer (50 mM Tris–HCl, 1% SDS, 10 mM EDTA, pH 8.0). Followed by treatment with 10 μl proteinase K (Thermo Fisher) at 55°C for 30 min, de‐cross‐linking was carried out by adding 30 μl 5 M NaCl and incubating at 65°C overnight. The next day, DNA was recovered and contaminated RNA was digested with RNase A at 37°C for 30 min. After purification, 3–5 μg Hi‐C DNA was sheared with a Q800R3 sonicator (QSONICA) by using the following parameters: 25% amplitude, 15 s ON, 15 s OFF, pulse‐on time for 4.5 min, to achieve a fragment size shorter than 500 bp. Sonicated products were further purified with Ampure beads to recover fragments larger than 300 bp. To remove biotin from unligated DNA ends, the DNA was mixed with 0.5 μl 10 mM dTTP, 0.5 μl 10 mM dATP and 5 U T4 DNA polymerase (Thermo Fisher) in a 50 μl reaction volume, and incubated at 20°C for 30 min. After purification with Ampure beads, end repair and adaptor ligation were performed following the manual of NEBNext Ultra II DNA Library Prep Kit (NEB). Subsequently, the ligated DNA was affinity‐purified with Dynabeads MyOne Streptavidin C1 beads (Invitrogen) and converted into sequencing libraries by amplification with 12 PCR cycles. The libraries were sequenced on an Illumina Novaseq instrument with 2 × 150 bp reads.

Reads mapping to the TAIR10 genome with bowtie 2 (v.2.2.4), removal of PCR duplicates and reads filtering were performed as previously described (Liu *et al*., 2016). Hi‐C map normalization was performed by using an iterative matrix correction function in the R package ‘HiTC’ (Servant *et al*., 2012). For all Hi‐C maps, the iterative normalization process was terminated when the eps value, indicating the similarity between matrices in two consecutive correction steps, dropped below 1 × 10^−4^. The filtered Hi‐C reads were used to create hic files with the juicer tool for interactive Hi‐C map inspection (Durand *et al*., 2016).

Chromatin interactions showing statistical significance were detected using the FitHiC2 tool (Kaul *et al*., 2020). The analysis was conducted at a 2 kb resolution. Intrachromosomal contacts with genomic distances ranging from 4 to 100 kb were selected for statistical significance testing using the parameters ‐L 4000 ‐U 100000 ‐r 2000. Following FitHiC2 analysis, chromatin contacts with an adjusted *P*‐value (*q*‐value) below 0.01 were defined as chromatin loops and retained for subsequent analyses.

### BAC‐based Capture Hi‐C

BAC plasmids used in the FISH experiment were also employed to generate probes for capture Hi‐C. Probes were labeled with biotin‐16‐dUTP (11 093 070 910; Roche) by nick translation following the manufacturer's instructions (11 745 808 910; Roche). Capture Hi‐C was performed based on the Twist Bioscience Target Enrichment Standard Hybridization v2 Protocol (Twist Bioscience, South San Francisco, CA, USA), with minor modifications. A total of 56 μl of probe solution was prepared by pooling the labeled probes into 40 μl hybridization mix (Twist Standard Hyb and Wash Kit, 104 446; Twist Bioscience), achieving a final concentration of 0.2–0.3 ng μl^−1^ for each BAC. In parallel, 24 μl of library solution was prepared by mixing 500 ng of each indexed Hi‐C library (generated using NEBNext Ultra II DNA Library Prep Kit, NEB) with 1.2 μl of NEXTFLEX Universal blockers (NOVA‐5143231; Revvity, Lafayette, CO, USA). After denaturing at 95°C for 5 min, the probe solution was cooled on ice for 5 min, and the library solution was equilibrated to room temperature. The probe and library solutions were then combined, well‐mixed and split into two PCR tubes, followed by adding 30 μl of Hybridization Enhancer (Twist Standard Hyb and Wash Kit, 104 446; Twist Bioscience) on top of each 40 μl mixture. Hybridization was further performed at 70°C for 16 h. The next day, 70 μl of hybridization product was rapidly transferred into 200 μl of pre‐heated Binding buffer (104 446; Twist Bioscience) containing 10 μl of Dynabeads MyOne Streptavidin C1 Beads (Invitrogen) and incubated at 68°C for 5 min. After washing once with Standard Wash Buffer 1 (104 446; Twist Bioscience) at 68°C, and three times with Wash Buffer 2 (104 446; Twist Bioscience) at 48°C, the beads were resuspended and combined using 20 μl of 10 mM Tris–HCl (pH 8.0). Capture libraries were further amplified using 1 μl of the beads‐bound DNA as template with Illumina P5 (5′‐AATGATACGGCGACCACCGA‐3′) and P7 (5′‐CAAGCAGAAGACGGCATACGA‐3′) primers, corresponding to the standard sequencing adapters of the Hi‐C libraries, for 15 PCR cycles.

### Immunolabelling

Nuclei were isolated and sorted from fixed calli as described in the FISH in the ‘Materials and Methods’ section, and then resuspended in PBS and treated at 65°C for 30 min. Subsequently, the nuclei were mixed with RNase A and transferred onto Superfrost Ultra Plus Adhesion Slides (Thermo Fisher Scientific). After 1 h incubation at 37°C, nuclei attached on the slides were sequentially washed with a graded series of alcohol solutions (100, 95, 90, 80, 60, and 30%), followed by a 5 min incubation in PBS. For antigen retrieval, slides were immersed in sodium citrate buffer (10 mM sodium citrate, 0.05% TWEEN 20, pH 6.0) and boiled in a microwave oven at 700 W for 15 min. After post‐fixation in 4% formaldehyde, slides were briefly washed with PBS and dehydrated with a graded ethanol series (30, 60, 80, 90, 95, and 100%). Dried slides were blocked with blocking solution (5% BSA in 4× SSC with 0.2% TWEEN 20) for 10 min at room temperature. CRWN1 was detected using a rabbit‐derived endogenous antibody generated following a published protocol (Blunt *et al*., 2020; 1 : 2000 dilution), as described in the western blot in the ‘Materials and Methods’ section, followed by a goat anti‐rabbit Alexa Fluor 546‐conjugated antibody (A11071, 1 : 500 dilution; Invitrogen). Confocal images were obtained with a Zeiss LSM700 system and processed with fiji image J software.

## Results

### *emf2* calli exhibit altered transcriptomic profiles yet retain responsiveness during root induction

**Fig. 1:**
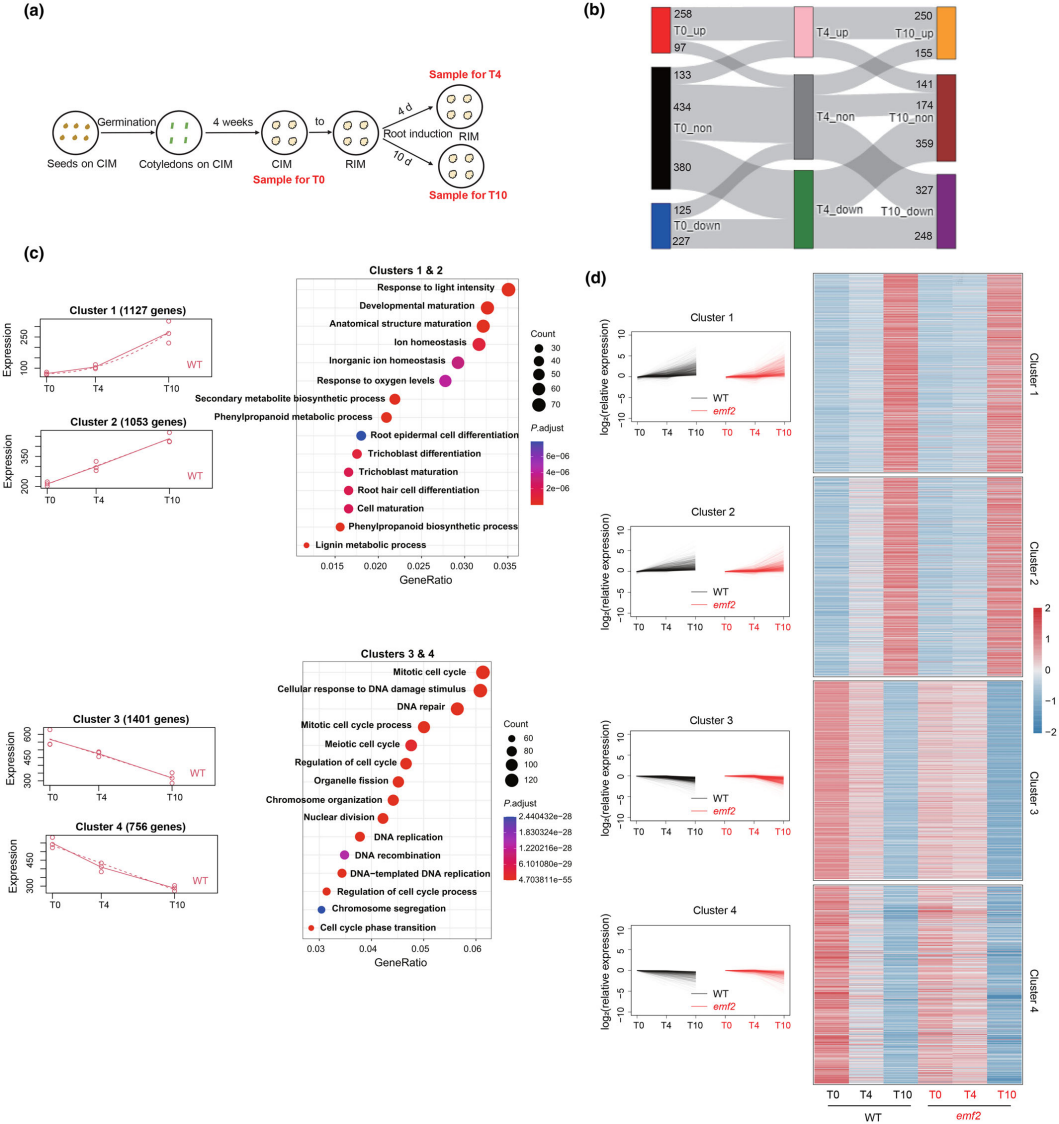
*EMF2* mutation alters transcriptomic profiles in Arabidopsis calli while retaining responsiveness during root induction. (a) The experimental setup. (b) Sankey plot illustrating differentially expressed genes in *emf2* compared with wild‐type at different time points. Numbers on the bundles represent the gene numbers of corresponding categories. (c) Genes exhibiting significant temporal expression changes in wild‐type were grouped into four clusters (left panels). Gene Ontology (GO) analysis for clusters 1 and 2 is displayed on the right top panel, while GO analysis for clusters 3 and 4 is shown on the right bottom. Dotted lines in the left panels denote regression curves. (d) Comparison of expression profiles of clustered genes between wild‐type and *emf2* during root induction. Left panels: expression patterns of clustered genes in wild‐type and *emf2* during root induction. Gene expression levels were normalized to the corresponding T0 to calculate relative expression values. Right panels: heat maps comparing the expression of clustered genes between wild‐type and *emf2* at different time points. Gene expression values were row‐scaled (mean‐centered and variance‐standardized), with the color scale indicating relative expression levels from low (blue) to high (red).

Our previous work showed that *emf2* was able to form calli on CIM, but failed to regenerate roots on RIM (Mandel *et al*., 2025), which aroused our curiosity about the ability of *emf2* to perceive induction signals and reprogramming gene expression during root induction. To address this question, we implemented a design with three time points to compare the gene expression between *emf2* and wild‐type during root regeneration. After germination on CIM, cotyledons from both wild‐type and *emf2* were trimmed and cultured on CIM for 4 wk, and the resulting calli harvested before root induction were designated as samples for time 0 (T0). Subsequently, the calli were transferred to RIM and harvested after 4 and 10 d, corresponding to time 4 (T4) and time 10 (T10), respectively. These time points represent the transition from a pluripotent cell state to the earliest committed state (T4) and the stage of active organogenesis (T10) (Fig. 1a). Next, we performed RNA sequencing (RNA‐seq), and principal component analysis (PCA) demonstrated good consistency among replicates (Fig. S1a). DEGs were further identified with the criteria log_2_(FC) > 1.6 and *q* < 0.01. At T0, 355 genes were upregulated in *emf2* and 352 genes were downregulated (Figs 1b, S1b; Table S2), indicating the differences between wild‐type and *emf2* calli before root induction. The number of misregulated genes in *emf2* at T4 and T10 was 998 (391 upregulated and 607 downregulated) and 980 (405 upregulated and 575 downregulated), respectively (Figs 1b, S1b), among which 188 and 122 genes showed consistent upregulation and downregulation across all the time points (Figs 1b, S1c). Additionally, most upregulated genes (258 out of 355 genes) in *emf2* T0 samples maintained their transcriptional changes on a later time point (i.e. T4 on RIM), and similar trend could also be observed from downregulated genes (227 out of 352 genes), suggesting that the impact of *emf2* mutation on transcriptome is similar for calli growing on CIM and RIM (Figs 1b, S1b). Furthermore, Gene Ontology (GO) enrichment analysis revealed that the genes upregulated at all time points were strongly associated with floral organ development (Fig. S1d), consistent with our previous findings that several MADS‐box transcription factors, as key regulators of developmental phase transitions, were significantly upregulated in *emf2* calli (Mandel *et al*., 2025) (Fig. S1e,f). In summary, these results indicate that *EMF2* mutation leads to persistent misregulation of gene expression throughout the root regeneration process.

To further investigate how callus cells respond to root induction, genes with significant temporal expression changes in wild‐type were identified and categorized into four clusters based on their expression patterns during the induction process (Fig. 1c; Table S3). Clusters 1 and 2 consisted of genes whose expression progressively increased as induction progressed, with cluster 2 showing a more linear trajectory. By contrast, genes in clusters 3 and 4 exhibited a continuous decrease in expression during induction. GO enrichment analysis revealed that genes in clusters 1 and 2 were predominantly associated with developmental maturation and differentiation, while clusters 3 and 4 were primarily involved in cell cycle and DNA replication (Fig. 1c). These results demonstrated a cell fate transition from cell proliferation to cell differentiation during the process of root induction from callus.

To evaluate whether *emf2* can respond to root induction, we further compared the transcriptomic profiles between wild‐type and *emf2* during the induction process. PCA of the RNA‐seq data revealed a clear distinction between genotypes, and the progression from T0 to T10 was similar in both wild‐type and *emf2*, implying that both may respond to root induction in a comparable manner (Fig. S1a). We then analyzed the expression of genes in each of the above‐mentioned clusters in *emf2* and found that the four clusters displayed similar expression patterns in both genotypes (Fig. 1d). Moreover, while several DNRR marker genes displayed expression level changes in *emf2*, their overall induction trajectories closely mirrored those observed in the wild‐type (Fig. S2a). Taken together, our data indicate that *emf2* mutants exhibit a generally comparable transcriptomic response to induction signals; however, the altered regulation of specific genes in *emf2* may underlie its inability to regenerate roots during induction.

### *EMF2* mutation leads to widespread reduction and focal increases of H3K27me3 in Arabidopsis calli

**Fig. 2:**
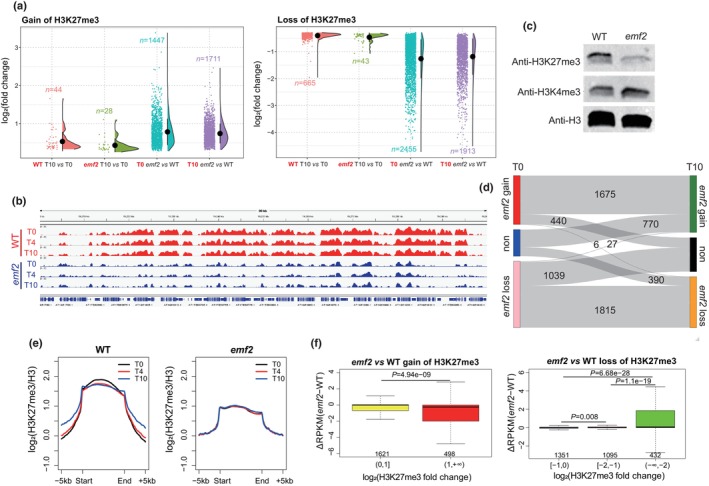
Disruption of *EMF2* leads to a large‐scale reduction of H3K27me3 in Arabidopsis calli. (a) Overview of differentially enriched sites (DESs) of H3K27me3 between samples (gain of H3K27me3: log_2_ enrichment fold change > 0, FDR < 0.05; loss of H3K27me3: log_2_ enrichment fold change < 0, FDR < 0.05). The violin plots illustrate the distribution of values for each group. Black dots represent the mean values, and the error bars indicate the SD. (b) Snapshot from IGV showing H3K27me3 levels within a 100 kb region across different samples. (c) Western blot displaying the abundance of histone modifications in wild‐type and *emf2* calli. (d) Sankey diagram showing genes associated with DESs at T0 and T10. Numbers on the flows represent the number of genes in each category. (e) Comparison of H3K27me3 profiles between wild‐type and *emf2* during root induction. (f) Comparison of gene expression between wild‐type and *emf2* at H3K27me3 differentially enriched sites. The box plots indicate the median (line within the box), the lower and upper quartiles (box), margined by the largest and smallest data points that are still within the interval of 1.5 times the interquartile range from the box (whiskers). *P* values indicate the two‐sided Mann–Whitney *U*‐test results.

As EMF2 is a key component of the PRC2 complex that catalyzes H3K27me3, we performed ChIP‐seq experiments to compare H3K27me3 profiles between wild‐type and *emf2* during root regeneration. Differentially enriched sites (DESs) of H3K27me3 were identified with a threshold of FDR < 0.05 and categorized into two groups: gain of H3K27me3 (with log_2_ enrichment fold change > 0) and loss of H3K27me3 (with log_2_ enrichment fold change < 0) (Table S4). As expected, the *emf2* mutation resulted in a large‐scale reduction of H3K27me3, reflected by a greater number of sites exhibiting a loss of H3K27me3 (Fig. 2a). Global changes in H3K27me3 modification in *emf2* were clearly evident in the genome browser, where H3K27me3 signals were strikingly decreased but not completely lost in *emf2* (Figs 2b, S3), as well as in the western blot assay (Fig. 2c).

By comparing the T10 samples to the corresponding T0 samples of the same genotype, we could assess the dynamics of H3K27me3 in response to root induction (Fig. S3a). In wild‐type calli, 665 sites exhibited lower H3K27me3 (log_2_ enrichment fold change < 0, FDR < 0.05) after induction, while 44 sites showed an increase (log_2_ enrichment fold change > 0, FDR < 0.05), indicating that the removal of H3K27me3 predominated the dynamics of this histone mark during root regeneration (Fig. 2a). On the contrary, only 71 DESs (43 loss and 28 gain) were identified during the course of root induction in *emf2* (Fig. 2a), suggesting attenuated H3K27me3 dynamics in the mutant. To further compare H3K27me3 changes between wild‐type and *emf2* during root induction, we identified genes associated with H3K27me3 DESs in *emf2* at T0 and T10 (Table S4). In total, 2878 genes showed reduced H3K27me3 at T0 in *emf2*, while 2119 genes exhibited increased levels. Notably, *c.* 70% of these genes (3490 out of 4997) maintained their differential H3K27me3 states at T10, indicating that most differences between wild‐type and *emf2* are stable throughout the root induction process (Fig. 2d). Further, the difference in H3K27me3 levels between wild‐type and *emf2* calli was more pronounced than the variations seen across different time points within the same genotype (Figs 2a,b, S3b,c), indicating that *emf2* exhibits a distinct H3K27me3 profile compared to wild‐type, rather than showing dynamic alterations during root induction. Interestingly, *c.* 1500 genomic loci with modest gain of H3K27me3 in *emf2* were identified, suggesting active H3K27me3 deposition by PRC2 complexes that did not contain EMF2 (Figs 2a, S3d). We further examined H3K27me3 profiles on genes with their flanking 5 kb regions. For genes that were enriched with H3K27me3 at T0 in wild‐type calli, the occupancy of H3K27me3 on the gene bodies decreased slightly after root induction; however, the H3K27me3 level of these genes was dramatically lower in *emf2* calli (Fig. 2e). In summary, our data revealed that wild‐type calli mainly undergo H3K27me3 removal rather than an increase at many loci during root induction, which does not occur to *emf2* calli. Instead, *emf2* calli exhibit a distinct and overall reduced H3K27me3 landscape.

To investigate the responsiveness of *emf2* to root induction concerning H3K27me3 dynamics, we identified genes located within the 665 DESs that exhibited reduced H3K27me3 levels at T10 compared with T0 in wild‐type (Fig. 2a; Table S4) and analyzed their H3K27me3 levels in *emf2*. Analysis of these genes revealed a similar downward trend in H3K27me3 levels at T10 relative to T0 in *emf2*, indicating that *emf2* largely followed the same trajectory as wild‐type, although the extent of reduction was less pronounced (Fig. S3e). Further examination of H3K27me3 levels at DNRR marker genes revealed that their expression trajectories are not closely associated with changes in H3K27me3 levels, suggesting that the regulation of these genes during root regeneration is largely independent of H3K27me3 dynamics (Fig. S2b). Altogether, by integrating the time‐course transcriptomic data, we conclude that *emf2* calli are able to respond to root induction at both the H3K27me3 and transcriptomic levels. However, the inability of *emf2* to maintain appropriate H3K27me3 and gene expression profiles before induction may contribute to its failure to regenerate roots.

### Changes in H3K27me3 have a limited impact on gene expression in *emf2* calli

Next, we analyzed the influence of dynamic H3K27me3 on gene expression in calli. At both T0 and T10, only a minority of *c.* 5% of genes (253 out of 4976 at T0; 298 out of 4662 at T10) with altered H3K27me3 levels in *emf2* were differentially expressed (Fig. S3g). Genes that exhibited both loss of H3K27me3 and upregulation in *emf2* were enriched for functions related to floral organ development (Fig. S3h), suggesting that their derepression may reflect a shift in cell identity in *emf2* calli. Considering that the extent of H3K27me3 enrichment may differentially affect gene expression, we further categorized genes into subgroups based on their fold change in H3K27me3 levels (Fig. 2f). As H3K27me3 profiles remained comparable across the three time points during root regeneration, subsequent analyses were focused on comparisons between wild‐type and *emf2* at T0. Genes that gained H3K27me3 in *emf2* exhibited reduced expression compared with wild‐type, with a larger extent in gain of H3K27me3 correlating with stronger repression of gene expression (Fig. 2f, left panel), consistent with the known role of H3K27me3 as a repressive histone mark. Interestingly, for genes that showed a loss of H3K27me3 in *emf2*, more than 80% maintained expression levels similar to those in wild‐type, and only a small portion with H3K27me3 fold change greater than four times exhibited increased expression in *emf2* (Fig. 2f, right panel). Further analysis of these genes in wild‐type revealed that those experiencing a loss of H3K27me3 in *emf2* showed relatively low expression in a wild‐type background (Fig. S3f). Collectively, our findings suggest that although the mutation of *EMF2* caused a global reduction of H3K27me3 in Arabidopsis calli, this decrease in the repressive mark is insufficient to fully derepress most of the affected genes.

### New long‐range chromatin interactions were formed in *emf2*

**Fig. 3:**
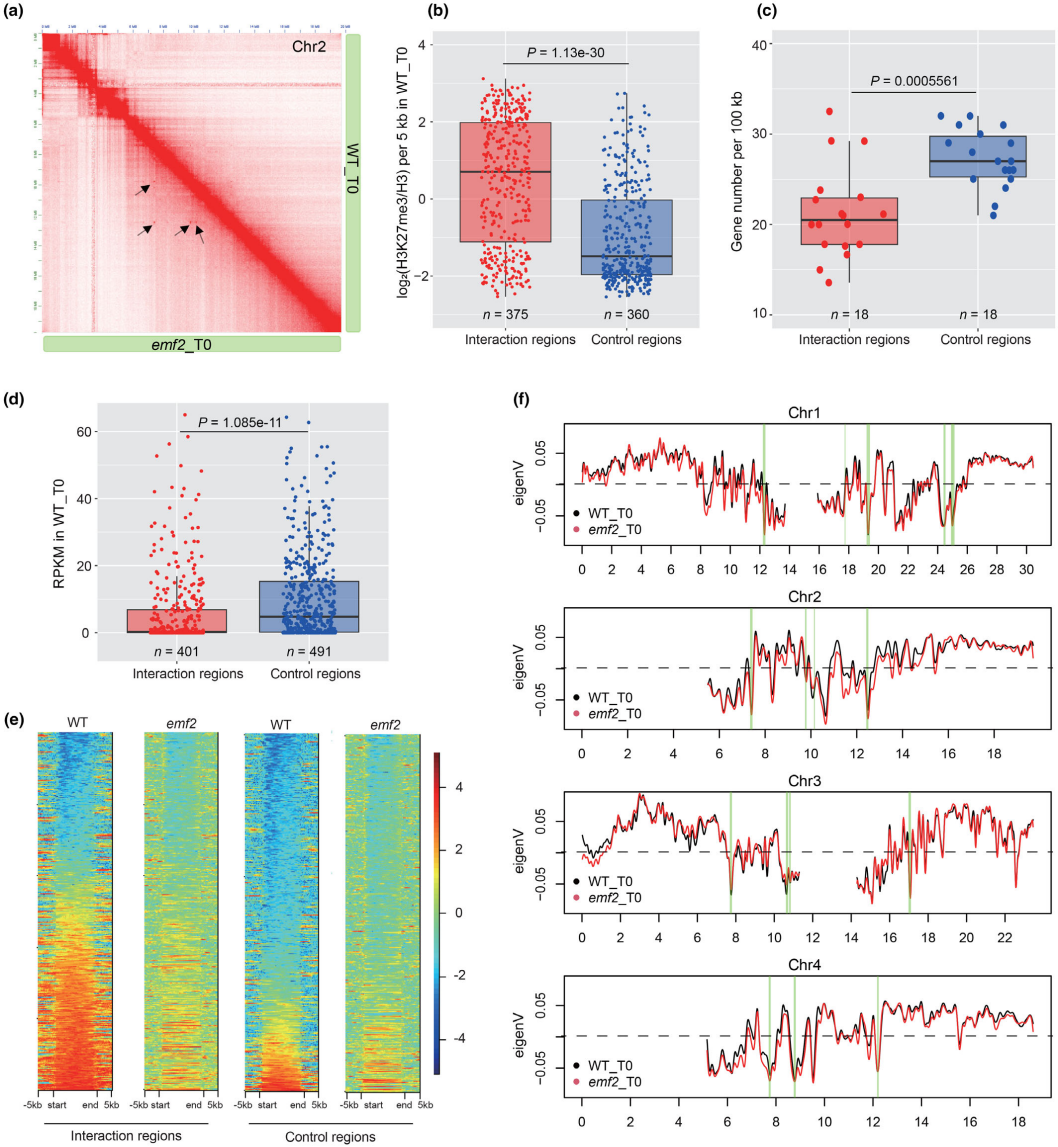
New long‐range chromatin interactions are observed in *emf2*. (a) Hi‐C map comparing wild‐type and *emf2* on chromosome 2, highlighting the new long‐range chromatin interactions in *emf2*. Arrows point to the interaction regions. The empirical *P* values are 9.99e−07, calculated from 10^6^ simulations comparing contacts between wild‐type and *emf2*. (b) Comparison of H3K27me3 levels between interaction regions and control regions in 5 kb windows in wild‐type at T0. (c) Comparison of gene densities between the identified interaction regions and control regions. (d) Expression levels of genes located within interaction regions (red) and control regions (blue) in wild‐type at T0. (e) Comparison of H3K27me3 levels between genes located in interaction regions and control regions at T0. The color bar represents the range of log_2_ (H3K27me3/H3) values from low (blue) to high (red). (f) Newly formed long‐range interactions in *emf2* are located in the B compartment. The green stripes show the *emf2*‐specific interaction regions. The box plots in (b–d) indicate the median (line within the box), the lower and upper quartiles (box), margined by the largest and smallest data points that are still within the interval of 1.5 times the interquartile range from the box (whiskers). *P* values indicate the two‐sided Mann–Whitney *U*‐test results.

It arouses our great interest to uncover the mechanisms underlying the maintenance of gene expression in *emf2*, despite the loss of H3K27me3. As H3K27me3 is known to play a role in 3D chromatin organization, and genes anchored within the same sub‐domains tend to be co‐regulated (Liu *et al*., 2016; Szabo *et al*., 2019; Huang *et al*., 2021), we performed *in situ* Hi‐C experiment to explore the chromatin architecture in *emf2*. Hi‐C maps were generated for both wild‐type and *emf2* across three time points during root induction (Fig. S4a). At the chromosomal scale, visual inspection of the Hi‐C maps did not reveal remarkable changes over the time‐course within each genotype, consistent with the limited alterations detected by the A/B compartment analysis (Fig. S4b), suggesting that the overall intrachromosomal contact patterns remain largely stable. Interestingly, comparison of intrachromosomal Hi‐C maps between wild‐type and *emf2* revealed newly formed long‐range chromatin interactions in *emf2* across nearly all chromosomes (Figs 3a, S5a; Table S5). Although these newly established interactions were relatively weak, they persisted in *emf2* at all three time points (Fig. S5b). To more rigorously define *emf2*‐specific interaction regions, we performed 10^6^ simulation tests to exclude regions that appeared as loop‐like structures in Hi‐C maps but did not have statistical significance relative to wild‐type. Notably, among the 18 annotated regions participating in the newly established long‐range interactions, six functioned as interaction hubs (i.e. 12.41–12.51 Mb on chromosome 2, and 7.68–7.80 Mb on chromosome 3), contacting with two to three regions at the same chromosome (Table S5). Furthermore, four of these hub regions were also involved in newly formed inter‐chromosome interactions in *emf2* (Fig. S6; Table S5), suggesting a broader reorganization of the 3D genome architecture in *emf2*. In Arabidopsis, the KNOT structure has been described as a higher‐order chromatin organization involving interactions among genomic regions across multiple chromosomes (Grob *et al*., 2014). Comparison of *emf2*‐specific interaction regions with KNOT ENGAGED ELEMENT (KEE) regions revealed no overlap, indicating that these newly identified interaction regions are not associated with the canonical KNOT architecture.

We next examined the expression patterns of genes located within these *emf2*‐specific long‐range interaction regions (IRs; regions involved in the establishment of *emf2*‐specific interactions). In total, 401 protein‐coding genes were identified (Table S5). To further explore their expression, these genes were categorized into three groups based on their expression levels in wild‐type at T0: silenced or inactive genes (RPKM < 1), intermediately expressed genes (1 < RPKM < 10), and active genes (RPKM > 10). More than half of these genes (*n* = 237) were inactive in wild‐type and showed similar expression in *emf2*. Likewise, the expression levels of both intermediately and actively expressed genes showed no significant differences between wild‐type and *emf2* (Fig. S7a). We further examined their expression during the root induction process. Across the T0 to T10 time points, genes located within IRs displayed similar expression levels in wild‐type and *emf2*, without obvious time‐dependent changes (Fig. S7b). Interestingly, three distinct gene clusters were found within IRs, which were *IAN1* to *IAN9* (from immune‐associated nucleotide‐binding protein gene family), *SCPL8* to *SCPL13* (from serine carboxypeptidase‐like protein family), and *CYP705A* and *CYP702A* gene families (Fig. S7c, top panel). These clustered genes also exhibited comparable expression between wild‐type and *emf2* and showed no clear changes during root induction (Fig. S7c, bottom panel). Altogether, these results indicate that although these genes are located within *emf2*‐specific IRs, their expression levels remain largely unchanged in *emf2* during root induction.

Manual inspection of the *emf2*‐specific IRs revealed that they were densely enriched with H3K27me3 in wild‐type calli, but exhibited a drastic reduction of this histone modification in *emf2* (Fig. S7d). To further characterize these regions, we compared them with randomly withdrawn regions in the genome. The annotated 18 IRs, with a median length of 100 kb (Fig. S7e; Table S5), alongside 18 regions of comparable lengths, selected randomly from chromosome arms, were used for comparison. In 5 kb window analyses, IRs exhibited significantly higher H3K27me3 levels in wild‐type at T0, consistent with their dense occupancy observed in the genome browser (Figs 3b, S7d). We also noticed a lower density of protein‐coding genes in IRs (Fig. 3c). Furthermore, the expression levels of genes located within the IRs were significantly lower than those in control regions in wild‐type at T0, correlating with a higher fraction of H3K27me3‐marked genes in IRs as opposed to control regions (Fig. 3d,e). Collectively, *emf2*‐specific IRs were characterized as relatively gene‐poor, large‐sized domains with high levels of H3K27me3 in wild‐type calli. Interestingly, these long‐range contacts were not observed on Hi‐C maps derived from *clf swn* calli (Feng *et al*., 2014), which are expected to have extensive H3K27me3 reduction due to the loss of key components of the PRC2 responsible for H3K27me3 deposition (Yin *et al*., 2023) (Fig. S8), implying that the altered patterns of IRs in different PRC2 mutants are closely associated with the specific ways in which H3K27me3 is deposited or lost in each genetic background.

### Spatial compartmentalization of *emf2*‐specific IRs

Chromatin organization within the nucleus, including Arabidopsis, is highly structured, forming regions that are spatially linked or separated. A binary annotation strategy is commonly used to categorize chromatin regions into two spatial compartments, termed ‘A’ and ‘B’ compartments. The A compartment is enriched with actively transcribed chromatins, whereas the B compartment contains more densely packed inactive regions (Lieberman‐Aiden *et al*., 2009; Feng *et al*., 2014). We found that the *emf2*‐specific IRs were exclusively located within the B compartment, despite the *EMF2* mutation not significantly altering the identity of chromatin compartmentalization (Fig. 3f).

**Fig. 4:**
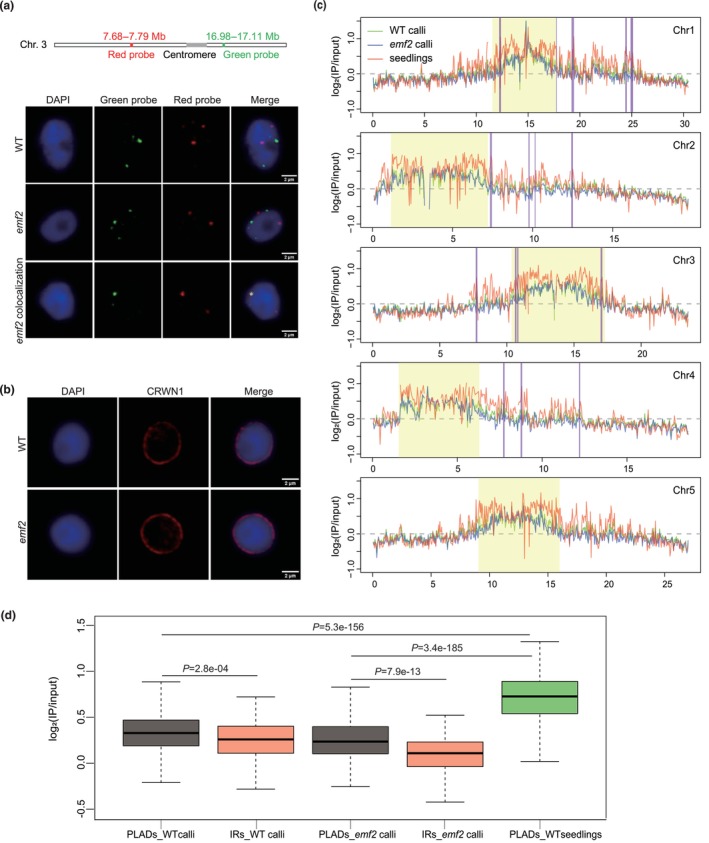
Association between *emf2*‐specific long‐range chromatin interaction regions and the nuclear periphery. (a) Fluorescent in situ hybridization (FISH) images showing the localizations of two regions involved in the establishment of an *emf2*‐specific interaction on chromosome 3 in wild‐type and *emf2* callus nuclei. The sketch shown above depicts the locations of the probed regions. Bars, 2 μm. (b) Immunohistostaining images depicting the localization of CRWN1 in Arabidopsis callus nuclei. Bars, 2 μm. (c) CRWN1‐chromatin association profiles in Arabidopsis seedlings, wild‐type calli, and *emf2* calli. Shaded yellow areas indicate pericentromeric regions, and the purple stripes show the *emf2*‐specific interaction regions. (d) Comparison of CRWN1 enrichment at PLADs and IRs among wild‐type calli, *emf2* calli, and seedlings. Box plots show the median (line within the box), the lower and upper quartiles (box), margined by the largest and smallest data points that are still within the interval of 1.5 times the interquartile range from the box (whiskers). *P* values indicate the two‐sided Mann–Whitney *U*‐test results.

Our previous work revealed that the spatial distribution of Arabidopsis A/B compartment exhibits a radial gradient, with chromatin from the B compartment being enriched at the nuclear periphery (NP) (Bi *et al*., 2017). We selected two regions on chromosome 3 (16.98–17.11 Mb, detected by green probes; 7.68–7.79 Mb, detected by red probes), which form a newly established long‐range interaction in *emf2*, to investigate their spatial location in callus nuclei using FISH. We defined a nucleus as NP‐localized if at least one green or red signal was detected at the NP. Based on this criterion, more than 80% of the analyzed nuclei (29/32 in wild‐type and 28/33 in *emf2*) exhibited NP‐localized signals for at least one locus, while over 35% (13/32 in wild‐type and 13/33 in *emf2*) showed NP‐localized signals for both loci, with similar patterns observed between wild‐type and *emf2* (Fig. 4a). Among the 33 *emf2* nuclei analyzed, two exhibited colocalization of the green and red signals (Fig. 4a, bottom panels); on the other hand, none of the 32 examined wild‐type calli nuclei showed signal colocalization (*P* value was 0.49 based on Fisher's exact test). Altogether, these results suggested that genomic regions involved in *emf2*‐specific long‐range interactions preferentially localize to the NP in Arabidopsis callus nuclei.

The plant lamin‐like protein CRWN1 is localized at the NP and mediates the tethering of B compartment chromatin to form plant lamina‐associated domains (PLADs) (Hu *et al*., 2019). In mammalian cells, an antagonistic relationship between H3K27me3 and genome‐lamina association has been reported (Siegenfeld *et al*., 2022; Guerreiro *et al*., 2024), raising the question of whether a similar mechanism operates in plants. To investigate this, we examined CRWN1‐chromatin association in wild‐type and *emf2* calli. Since CRWN1 exhibits dynamic localization (Wang *et al*., 2023), we first assessed whether CRWN1 proteins had different nuclear localization patterns in Arabidopsis calli. With an endogenous CRWN1 antibody that specifically detected CRWN1 (Fig. S9), immunolabelling revealed that CRWN1 predominantly localized at the NP in calli, consistent with its pattern in Arabidopsis seedlings, and its localization remained unchanged in *emf2* (Fig. 4b).

Subsequently, we examined CRWN1‐chromatin association via chromatin immunoprecipitation sequencing (ChIP‐seq). Similar to seedlings, both wild‐type and *emf2* calli tissues exhibited CRWN1‐chromatin contacts in pericentromeric regions; moreover, on a genomic scale, the wild‐type and *emf2* calli showed highly similar CRWN1‐chromatin interaction patterns, including *emf2*‐specific IRs (Fig. 4c). Notably, PLADs exhibited significantly weaker CRWN1‐chromatin interactions in wild‐type and *emf2* calli compared to those observed in seedlings (Fig. 4c,d). Further analysis of CRWN1 enrichment at IRs, approximately half of which overlap with PLADs (1.04 Mb out of 2.22 Mb), revealed CRWN1 ChIP signals that were comparable to but overall lower than those observed at PLADs. Interestingly, this reduction was more pronounced in *emf2* calli (Fig. 4d). Altogether, these results suggest distinct mechanisms in plants and animals. In totipotent mammalian embryo cells, loss of H3K27me3 in PRC2 knockout cells is associated with increased genome‐lamina interactions (Guerreiro *et al*., 2024), whereas IRs‐lamina interactions are reduced in *emf2*, where IRs exhibit a substantial loss of H3K27me3.

### Fine‐scale chromatin interaction profiles of *emf2*‐specific IRs

**Fig. 5:**
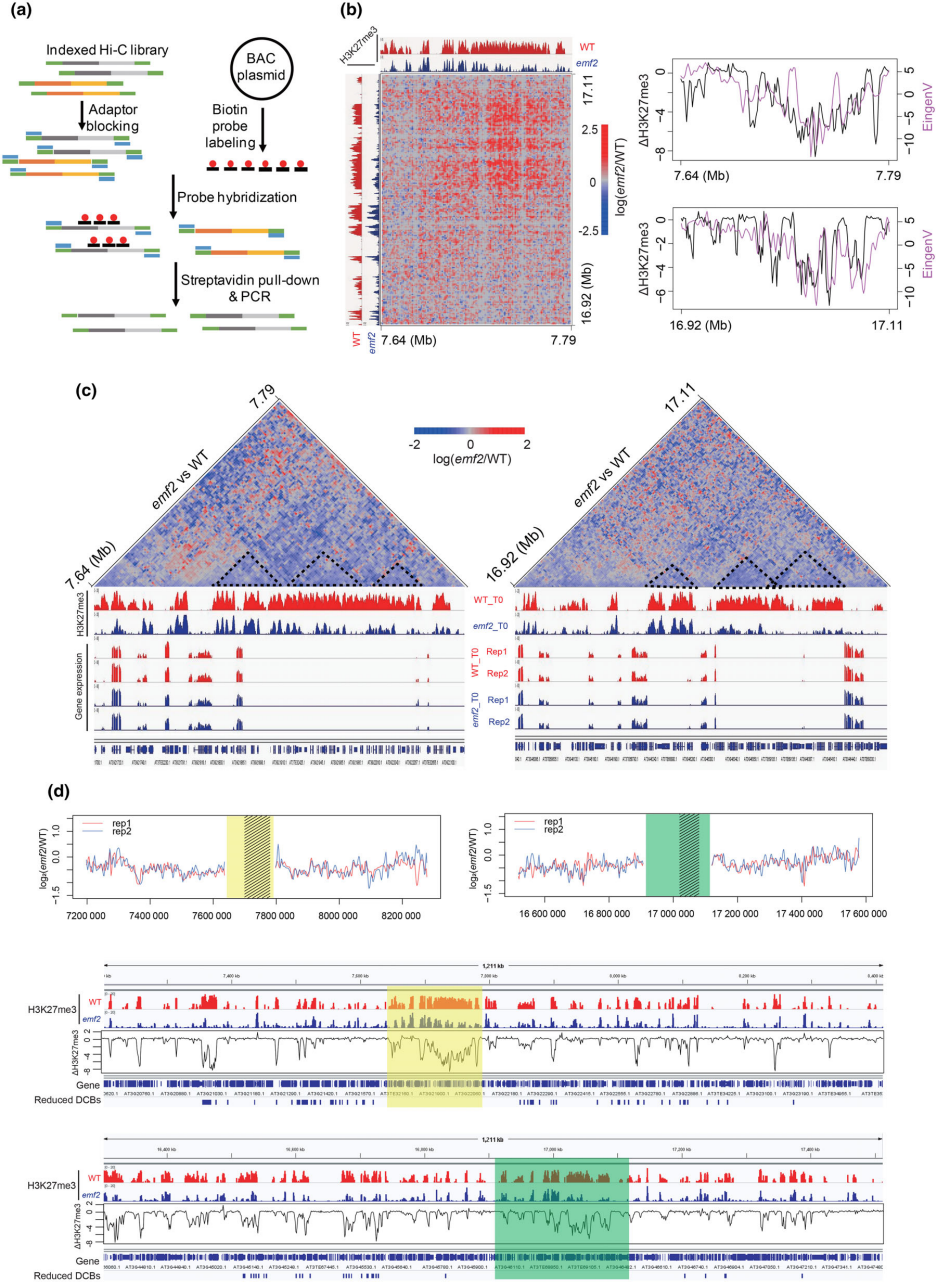
Fine‐scale chromatin interaction profiles within and around *emf2*‐specific interaction regions. (a) An overview of the bacterial artificial chromosome (BAC)‐based Capture Hi‐C workflow. Indexed Hi‐C libraries are hybridized with biotin‐labeled probes generated from BAC plasmids after adaptor blocking. Target fragments are captured using streptavidin beads, and the captured libraries are amplified with PCR for sequencing. (b) Left panel: Comparison of BAC‐based Capture Hi‐C contact between *emf2* and wild‐type (WT) at the *emf2*‐specific IRs (7.64–7.79 Mb and 16.92–17.11 Mb on chromosome 3) showing the core regions involved in long‐range interactions in *emf2*. H3K27me3 levels of the corresponding regions are included. Colors indicate the difference in chromatin interaction strengths, expressed as the logarithm of the ratio between *emf2* and wild‐type. Right panels: Bin‐by‐bin comparison of ΔH3K27me3 and the first component (PC1) derived from principal component analysis (PCA) of the differential interaction matrix (*emf2* vs WT) across the interaction regions (IRs) shown in the left panel. Black lines represent ΔH3K27me3, and purple lines represent PC1 values. (c) Comparison of intra‐IR contacts between *emf2* and WT. The levels of H3K27me3 and gene expression are included. Colors indicate the difference in chromatin interaction strengths as in (b). Triangles in the Hi‐C maps mark the regions exhibiting reduced intra‐IR interactions in *emf2*. (d) BAC‐based Capture Hi‐C analysis of interactions between core‐IRs and adjacent regions. Top: comparison of chromatin contacts between peri‐IRs and core‐IRs in *emf2* and wild‐type. Bottom: IGV tracks showing H3K27me3 levels in the analyzed regions in wild‐type and *emf2*, together with an additional track exhibiting ΔH3K27me3 (*emf2* – WT). Differential‐contact bins (DCBs) displaying reduced interaction with core‐IRs are indicated. Yellow and green rectangles represent BAC‐covered regions, and the black strips denote the core‐IRs. In the upper plots, BAC‐covered regions (including core‐IRs) are excluded from the quantification and are therefore not displayed.

To further explore chromatin contact patterns within and around the *emf2*‐specific IRs, we developed a BAC (bacterial artificial chromosome)‐based Capture Hi‐C method. In this approach, regions of interest were enriched from indexed Hi‐C libraries using biotin‐labeled probes generated from BAC plasmids (Fig. 5a). Using BAC probes that covered a pair of IRs on chromosome 3 (7.68–7.79 Mb and 16.98–17.11 Mb), we could achieve over 100‐fold enrichment of these regions with our BAC‐based Capture Hi‐C method (Fig. S10a). Noticeably, after hybridization enrichment, the fraction of Hi‐C reads concerning IRs increased from *c.* 0.2% to over 20%, and we found no hybridization biases across samples (Fig. S10a,b). As a result, this BAC‐based Capture Hi‐C approach enabled us to analyze chromatin contact patterns at sub‐kilobase resolution. We found that the core interaction regions (core‐IRs) mediating *emf2*‐specific long‐range interactions display dense and strong H3K27me3 modification in wild‐type, but show a dramatic reduction of this mark in *emf2* (Fig. 5b, left panel). Bin‐by‐bin comparison of *emf2* to wild‐type further demonstrated that the reduction of H3K27me3 is closely associated with changes in chromatin interaction patterns within the core‐IRs (Fig. 5b, right panels). While establishing new long‐range interactions, the loss of H3K27me3 in *emf2* weakened intra‐IR contacts without obvious changes in gene expression (Fig. 5c). For each core‐IR, we further examined 500 Kb upstream and downstream from BAC‐covered regions (adjacent regions), and analyzed their contacts with the core‐IR (Fig. 5d). We identified differential‐contact bins (DCBs) that exhibited altered contact frequency with the core‐IR between wild‐type and *emf2*, based on the following criteria: (1) bins with more than 20 Hi‐C contacts with the core‐IR; (2) bins showing at least a 1.5‐fold difference in contact frequency between wild‐type and *emf2*; and (3) these differences were reproducible in both replicates. In total, 64 and 29 DCBs were identified in the adjacent regions of the two IRs, respectively. Interestingly, all DCBs exhibited reduced contact with the corresponding core‐IR in *emf2*, and most of them showed high H3K27me3 levels in wild‐type, which were markedly reduced in *emf2* (Fig. 5d). Altogether, using a BAC‐based Capture Hi‐C approach, we achieved fine‐scale mapping of chromatin interactions within and around IRs. These results show that the loss of H3K27me3 in *emf2* calli is associated with weakened intra‐ and peri‐IR interactions, accompanied by chromatin reconfiguration and the emergence of new long‐range interactions.

### Gene interactions in *cis* were associated with gene expression

The emergence of *emf2*‐specific IRs and their reshaped chromatin contacts prompted us to further characterize changes in chromatin organization in *emf2* calli. To this end, we analyzed Hi‐C data in wild‐type and *emf2* at T0 to identify chromatin loops. Intrachromosomal contacts spanning genomic distances of 4–100 kb were annotated in both genotypes (Table S6). Compared with wild‐type, *emf2* exhibited fewer chromatin loops (112 146 vs 85 046), with approximately half of the loops detected in wild‐type also present in *emf2* (Fig. S11a). Quantitative analysis revealed that genotype‐specific loops, particularly those unique to *emf2*, tended to display higher loop strength (Fig. S11b). To examine the relationship between chromatin architecture and H3K27me3, we assessed correlations between loop strength and H3K27me3 levels. Although genome‐wide correlations were weak (Fig. S11c), loops detected exclusively in wild‐type showed significantly higher H3K27me3 levels (Fig. S11d). Moreover, in wild‐type calli, loops within the top 10% of strength were associated with higher H3K27me3 signals (Fig. S11e). Together, these findings suggested that H3K27me3 contributes to chromatin contacts in Arabidopsis calli.

**Fig. 6:**
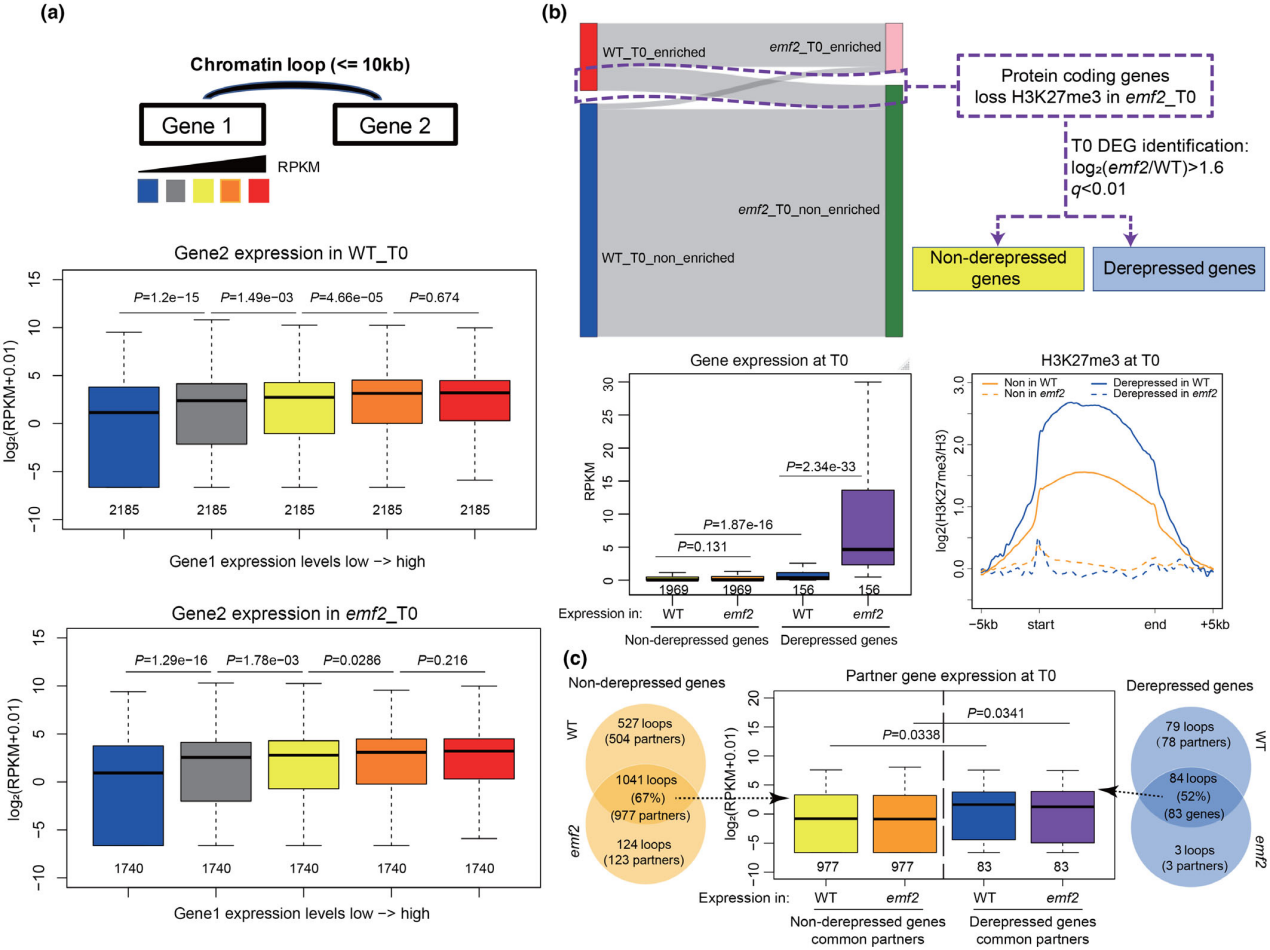
Association between gene interactions in *cis* and gene expression. (a) Genes with similar expression levels tend to form partnerships. Gene pairs forming loops shorter than 10 kb were grouped into five categories based on the expression level of ‘gene 1’, and the expression levels of ‘gene 2’ were examined in the corresponding genetic background. (b) Identification of derepressed and non‐derepressed genes (top panel). Expression (bottom left panel) and H3K27me3 levels (bottom right panel) of derepressed and non‐derepressed genes in wild‐type and *emf2* at T0. ‘Non’ in the bottom right panel is short for non‐derepressed genes. (c) Identification of common partner genes shared between wild‐type and *emf2* for both derepressed and non‐derepressed groups at T0, along with their expression levels in both wild‐type and mutant background. The box plots in (a–c) indicate the median (line within the box), the lower and upper quartiles (box), margined by the largest and smallest data points that are still within the interval of 1.5 times the interquartile range from the box (whiskers). *P* values indicate the two‐sided Mann–Whitney *U*‐test results.

To further assess the relationship between chromatin architecture and gene expression, we used chromatin loop annotation to identify interacting gene pairs. For each gene pair, the gene with a smaller coordinate in the reference genome was designated as ‘gene 1’ and the corresponding partner as ‘gene 2’ (Table S7). The ‘gene 1’ group was then divided into five subsets based on expression levels, and the expression of their partners (‘gene 2’) was subsequently examined. This analysis was performed for wild‐type and *emf2*, respectively. Interestingly, for gene pairs with distances shorter than 10 kb, regardless of genetic background, chromatin interactions preferentially occur between genes with concordant expression levels (Fig. 6a). A similar pattern was also observed from gene loops larger than 10 kb (Fig. S11f).

In *emf2*, most of the genes with reduced H3K27me3 levels remained transcriptionally repressed, with only a small number exhibiting upregulation (Figs 2f, S3f). To distinguish these genes whose ectopic expression may contribute to the phenotype of *emf2* and characterize their features, we classified protein‐coding genes with reduced H3K27me3 in *emf2* into two groups based on their expression changes. ‘Derepressed genes’ were defined by two criteria: (1) reduced H3K27me3 levels in *emf2* and (2) upregulation in *emf2* (log_2_(FC) > 1.6, *q* < 0.01). Protein‐coding genes exhibiting decreased H3K27me3 but not meeting the criteria for upregulated DEGs were classified as ‘non‐derepressed genes’ (Fig. 6b, top panel). Both groups were expressed at low levels or silenced in wild‐type; however, derepressed genes showed significantly higher basal expression (Fig. 6b, bottom left panel). Approximately 50% of non‐derepressed genes (980 of 1969) were completely silent in wild‐type (RPKM = 0), whereas only 10% of derepressed genes (16 of 156) were silent, indicating that most derepressed genes are weakly expressed rather than fully silenced. Comparative analysis further revealed substantially higher H3K27me3 levels across gene bodies of derepressed genes in wild‐type, accompanied by a pronounced loss of this modification in *emf2* calli (Fig. 6b, bottom right panel). Subsequently, we annotated chromatin loops spanning < 10 kb for derepressed and non‐derepressed genes to identify their partner genes. Overlap analysis was then performed to determine whether derepressed genes in *emf2* experienced changes in their local interaction partners, potentially facilitating interactions with actively expressed genes. Similar to non‐derepressed genes, for which 67% of loops identified in wild‐type (1041 of 1568) were retained in *emf2*, 52% of loops (84 of 163) associated with derepressed genes were shared between wild‐type and *emf2* (Fig. 6c venn diagrams). This suggests that derepressed genes did not undergo dramatic changes in their local interaction partners in *emf2*. We then examined the expression of partner genes within shared loops. Interestingly, in both genotypes, the partners of derepressed genes showed significantly higher expression levels compared with those of non‐derepressed genes (Fig. 6c, middle panel). In summary, our findings revealed that derepressed genes generally retain their interaction partners but display the following features: they exhibit basal transcriptional activities in wild‐type tissue, preferentially interact with transcribed genes via forming chromatin loops, and experience a substantial loss of H3K27me3 in *emf2* calli.

## Discussion

Eukaryotic development originates from a totipotent cell that undergoes successive rounds of division and differentiation to generate diverse cell types that function in a coordinated manner (Benfey, 2005). A central challenge in this process is the establishment and stable maintenance of cell identity, which provides the foundation for proper cellular differentiation and organismal development (Kim & Kingston, 2022). Polycomb group (PcG) proteins play a key role in this process by repressing incompatible or detrimental transcriptional programs through deposition of repressive histone marks and chromatin compaction (Xiao & Wagner, 2015). Since their initial identification as repressors of *Hox* genes in *Drosophila*, PcG proteins have been widely recognized as conserved regulators of cell fate determination across eukaryotes, including plants (Xiao & Wagner, 2015; Kassis *et al*., 2017).

In Arabidopsis, EMF2 is required to maintain vegetative identity by repressing floral developmental programs (Moon *et al*., 2003). Consistent with previous observations in seedlings, we found that *emf2* calli exhibit persistent activation of floral identity genes, and this ectopic transcriptional state is already established before root induction (Figs S1d, S3h). Although *emf2* calli fail to regenerate roots, they retain the capacity to respond to inductive signals at both the transcriptional level and in terms of H3K27me3 dynamics (Figs 1d, S3e). These findings suggest that the loss of regenerative competence is not due to a failure in signal perception, but rather reflects a disruption of cell identity that compromises the coordinated transcriptional programs required for organogenesis. Notably, callus formation itself is not impaired in *emf2*, highlighting the importance of future single‐cell approaches to resolve lineage‐specific transcriptional and epigenetic states during regeneration.

At a chromatin level, H3K27me3 is known to contribute to the formation of chromatin loops in Arabidopsis, and its dynamic changes are closely linked to chromatin reorganization (Liu *et al*., 2016; Huang *et al*., 2021). In this study, we identified reorganization of chromatin interactions in *emf2* calli, where novel long‐range chromatin interactions were established. Consistent with previous reports in *clf‐29* and *clf swn* mutants, regions involved in *emf2*‐specific long‐range interactions exhibited reduced H3K27me3 levels together with weakened local chromatin interactions (Fig. 5c) (Feng *et al*., 2014; Huang *et al*., 2021; Yin *et al*., 2023). However, in contrast to *clf‐29*, where loss of H3K27me3 is often associated with transcriptional activation and the establishment of new interactions with active chromatin regions (Huang *et al*., 2021), the majority of genes located within novel *emf2*‐specific IRs remained transcriptionally inactive despite substantial loss of H3K27me3. This observed decoupling between chromatin reorganization and transcriptional alteration suggests that changes in 3D genome architecture are not solely associated with gene expression dynamics. Instead, chromatin reconfiguration may also represent an alternative structural state that stabilizes transcriptional outputs under conditions of epigenetic perturbation. In this context, IRs in *emf2* may reflect a rebalanced chromatin organization that maintains gene repression even in the absence of canonical histone marks.

One potential explanation for this stability is the spatial compartmentalization of these regions. We found that IRs reside within the B compartment and are associated with the NP, largely mediated by the lamin‐like protein CRWN1. In both wild‐type and *emf2* calli, CRWN1 predominantly localizes to the NP and binds pericentromeric regions, consistent with its proposed role in tethering chromatin domains (Hu *et al*., 2019). The overall chromatin binding pattern of CRWN1 appears similar between callus and seedling tissues, despite their distinct developmental states, suggesting a conserved role of lamin in nuclear architecture maintenance as a scaffold protein. However, the strength of the CRWN1‐chromatin association is reduced in callus, particularly in *emf2*. Given that CRWN1 has been implicated in constraining chromatin mobility and anchoring lamina‐associated domains (Hu *et al*., 2019; Sakamoto *et al*., 2022; Yuan *et al*., 2025), this reduction may be associated with increased chromatin flexibility and the emergence of long‐range interactions in *emf2*. At the same time, the peripheral localization of these regions may contribute to maintaining a repressive environment, thereby buffering against transcriptional activation despite substantial loss of H3K27me3.

Consistent with this idea, we identified three gene clusters located within *emf2*‐specific IRs; the majority of them exhibit low expression in both wild‐type and *emf2* calli. These clusters include members of gene families that are typically organized in tandem arrays and associated with stress‐responsive and specialized metabolic pathways in plants (Lu *et al*., 2021; Xu *et al*., 2021; Marszalek‐Zenczak *et al*., 2023). Previous work has shown that some plant biosynthetic gene clusters are embedded within highly interactive chromatin domains that undergo dynamic conformational changes across organs. These structural transitions are associated with differences in transcriptional activity, as active and repressed clusters adopt distinct chromatin conformations (Nützmann *et al*., 2020). By contrast, the gene clusters identified here participate in *emf2*‐specific chromatin interactions without exhibiting corresponding transcriptional changes. This observation suggests two non‐mutually exclusive possibilities: chromatin reorganization may contribute to alternative modes of coordinated regulation among clustered genes, or coordinated transcriptional repression may be associated with the establishment of these long‐range interactions.

Mechanistically, PcG‐mediated repression is primarily mediated by two core complexes, PRC1 and PRC2. In a hierarchical model, PRC1‐catalyzed H2A monoubiquitination promotes PRC2 recruitment, which in turn deposits H3K27me3 (Blackledge *et al*., 2014; Kalb *et al*., 2014). Both H2A ubiquitination and preexisting H3K27me3 can enhance PRC2's methyltransferase activity, promoting the spreading of H3K27me3 across target loci (Xu *et al*., 2010; Kalb *et al*., 2014). As a repressive histone mark, one would naturally expect that the loss of this mark would lead to an increase in gene expression. However, only a small subset of genes were derepressed in *emf2* calli (Figs 2f, S3g). Further analysis revealed that these derepressed genes, associated with floral development, share distinct features: they exhibit basal transcriptional activity in wild‐type calli, preferentially engage in chromatin interactions with actively transcribed regions, and undergo pronounced H3K27me3 loss. These characteristics distinguish them from genes that remain transcriptionally stable, suggesting intrinsic differences in their regulatory potential.

Genome‐wide analyses of H2AK121ub and H3K27me3 distribution reveal partial overlap of these marks in Arabidopsis, with genes marked by both H2AK121ub and H3K27me3 showing higher average expression levels than those marked by H3K27me3 alone (Zhou *et al*., 2017; Kralemann *et al*., 2020). A model has been proposed in which chromatin regions marked by both H2AK121ub and H3K27me3 are transcriptionally responsive, whereas regions marked by H3K27me3 only, which are PRC1‐independent, remain transcriptionally inactive. This indicates that the presence of H2AK121ub allows genes to toggle between repression and activation through the deposition and removal of H3K27me3 (Kralemann *et al*., 2020; Baile *et al*., 2022). Based on this model, we hypothesize that differences in H2AK121ub level between derepressed and non‐derepressed genes may underlie their different behavior in Arabidopsis calli.

Studies in mammalian embryonic stem (ES) cells have shown that many PcG target genes are ‘bivalent’, carrying both repressive (H3K27me3) and active (H3K4me3) histone marks (Bernstein *et al*., 2006). ChIP‐seq analyses of PcG proteins and related modifications in ES cells identified two classes of bivalent genes: those bound by both PRC1 and PRC2, and those bound only by PRC2. Bivalent genes co‐occupied by PRC1 and PRC2 are resistant to H3K27me3 loss during differentiation, maintaining a highly conserved repressive chromatin state (Ku *et al*., 2008). Further investigation of PRC1 occupancy at H3K27me3 decreased loci in *emf2* will provide important insights into the coordination between PcG complexes in gene regulation during development. Furthermore, for some gene loci (e.g. *Hox* in mouse), PcG proteins may impose more spatial constraints, for which gene activation is accompanied by a sequential loss of H3K27me3 and a simultaneous increase in H3K4me3 (Soshnikova & Duboule, 2009). In support of this scenario, some of the genes marked with H3K27me3, and showing no H3K4me3 or expression in wild‐type calli, acquire H3K4me3 and are expressed upon *EMF2* mutation (Mandel *et al*., 2025). Additionally, a recent study in rice suggested that a threshold of the H3K27me3 : H3K4me3 ratio is important for gene regulation (Zhang *et al*., 2025), it would be of great interest to examine the H3K4me3 profiles of *emf2* during root regeneration.

In summary, our work underscores the critical role of EMF2‐mediated H3K27me3 in shaping 3D chromatin architecture and reveals its relationship with gene expression during plant regeneration.

## Competing interests

None declared.

## Author contributions

CL, LEW and ZW conceived and designed the experiments. ZW, MA, TM and CL conducted the experiments. ZW, LEW and CL analyzed the data. ZW, LEW and CL wrote the paper.

## Disclaimer

The New Phytologist Foundation remains neutral with regard to jurisdictional claims in maps and in any institutional affiliations.

## Supporting information


**Fig. S1** Overview of transcriptomic profiles between wild‐type and *emf2* during root regeneration.
**Fig. S2** Patterns of *de novo* root regeneration (DNRR) marker genes in wild‐type and *emf2* during root induction.
**Fig. S3** Comparison of H3K27me3 differentially enriched sites within and between genotypes during root regeneration.
**Fig. S4** Genome‐wide chromatin interaction analyses of wild‐type and *emf2* across different time points.
**Fig. S5** Comparisons of Hi‐C maps between wild‐type and *emf2* during root induction.
**Fig. S6** Hub regions involved in new long‐range chromatin interactions in *cis* form *trans* chromatin interactions in *emf2*.
**Fig. S7** Characteristics of the *emf2*‐specific long‐range interaction regions.
**Fig. S8** Comparison of Hi‐C maps between *emf2* and *clf swn*.
**Fig. S9** Detection of CRWN1 using an endogenous antibody by western blot.
**Fig. S10** Enrichment of target regions with BAC‐based Capture Hi‐C.
**Fig. S11** Relationship between loop strength, H3K27me3, and gene expression at T0.
**Fig. S12** Uncropped western blot images related to Fig. 2(c).
**Table S1** Information of bacterial artificial chromosomes (BACs) used in this work.

**Table S2** DEGs between *emf2* and wild‐type.

**Table S3** List of cluster genes during root induction.

**Table S4** Information of DESs identified between samples.

**Table S5** List of long‐range interaction regions and genes located within these regions.

**Table S6** Information of chromatin loops identified in wild‐type and *emf2*.

**Table S7** Information of gene pairs identified in wild‐type and *emf2* at T0.
Please note: Wiley is not responsible for the content or functionality of any Supporting Information supplied by the authors. Any queries (other than missing material) should be directed to the *New Phytologist* Central Office.

## Data Availability

Short read data of *in situ* Hi‐C, ChIP‐seq, and RNA‐seq are publicly available at NCBI Sequence Read Archive under accession no. PRJNA1238802. Large datasets, such as normalized Hi‐C matrices and ChIP‐seq BigWig track files, are available in the figshare repository. They are accessible with the following link: doi: 10.6084/m9.figshare.28607348.
