# Supplementary material for: EMF2 deficiency disrupts epigenetic and chromatin organization landscapes, blocking root regeneration competence in Arabidopsis
Source: New Phytol. 2026 May 28;251(4):1792–810. doi: 10.1111/nph.71298 (PMC13373812; doi:10.1111/nph.71298)
Supplement: Supplementary file 1 — Fig. S1 Overview of transcriptomic profiles between wild‐type and emf2 during root regeneration. Fig. S2 Patterns of de novo root regeneration (DNRR) marker genes in wild‐type and emf2 during root induction. Fig. S3 Comparison of H3K27me3 differentially enriched sites within and between genotypes during root regeneration. Fig. S4 Genome‐wide chromatin interaction analyses of wild‐type and emf2 across different time points. Fig. S5 Comparisons of Hi‐C maps between wild‐type and emf2 during root induction. Fig. S6 Hub regions involved in new long‐range chromatin interactions in cis form trans chromatin interactions in emf2. Fig. S7 Characteristics of the emf2‐specific long‐range interaction regions. Fig. S8 Comparison of Hi‐C maps between emf2 and clf swn. Fig. S9 Detection of CRWN1 using an endogenous antibody by western blot. Fig. S10 Enrichment of target regions with BAC‐based Capture Hi‐C. Fig. S11 Relationship between loop strength, H3K27me3, and gene expression at T0. Fig. S12 Uncropped western blot images related to Fig. 2(c). Table S1 Information of bacterial artificial chromosomes (BACs) used in this work. [file NPH-251-1792-s004.pdf]

New Phytologist Supporting Information

Article title: *EMF2* Deficiency Disrupts Epigenetic and Chromatin Organization Landscapes, Blocking Root Regeneration Competence in *Arabidopsis*

Authors: Zhidan Wang[1*], May Avraham[2], Tali Mandel[2], Leor Eshed Williams[2*], Chang Liu[1,3*]

Article acceptance date: 8 May 2026

The following Supporting Information is available for this article:

Fig. S1 Overview of transcriptomic profiles between wild-type and *emf2* during root regeneration.

Fig. S2 Patterns of *de novo* root regeneration (DNRR) marker genes in wild-type and *emf2* during root induction.

Fig. S3 Comparison of H3K27me3 differentially enriched sites within and between genotypes during root regeneration.

Fig. S4 Genome-wide chromatin interaction analyses of wild-type and *emf2* across different time points.

Fig. S5 Comparisons of Hi-C maps between wild-type and *emf2* during root induction.

Fig. S6 Hub regions involved in new long-range chromatin interactions in *cis* form *trans* chromatin interactions in *emf2*.

Fig. S7 Characteristics of the *emf2*-specific long-range interaction regions.

Fig. S8 Comparison of Hi-C maps between *emf2* and *clf swn*.

Fig. S9 Detection of CRWN1 using an endogenous antibody by Western blot.

Fig. S10 Enrichment of target regions with BAC-based Capture Hi-C.

Fig. S11 Relationship between loop strength, H3K27me3, and gene expression at T0.

Fig. S12 Uncropped Western blot images related to Fig. 2c.

Table S1 Information of bacterial artificial chromosomes (BACs) used in this work

Table S2 DEGs between *emf2* and wild-type

Table S3 List of cluster genes during root induction

Table S4 Information of DESs identified between samples

Table S5 List of long-range interaction regions and genes located within these regions

Table S6 Information of chromatin loops identified in wild-type and *emf2*

Table S7 Information of gene pairs identified in wild-type and *emf2* at T0

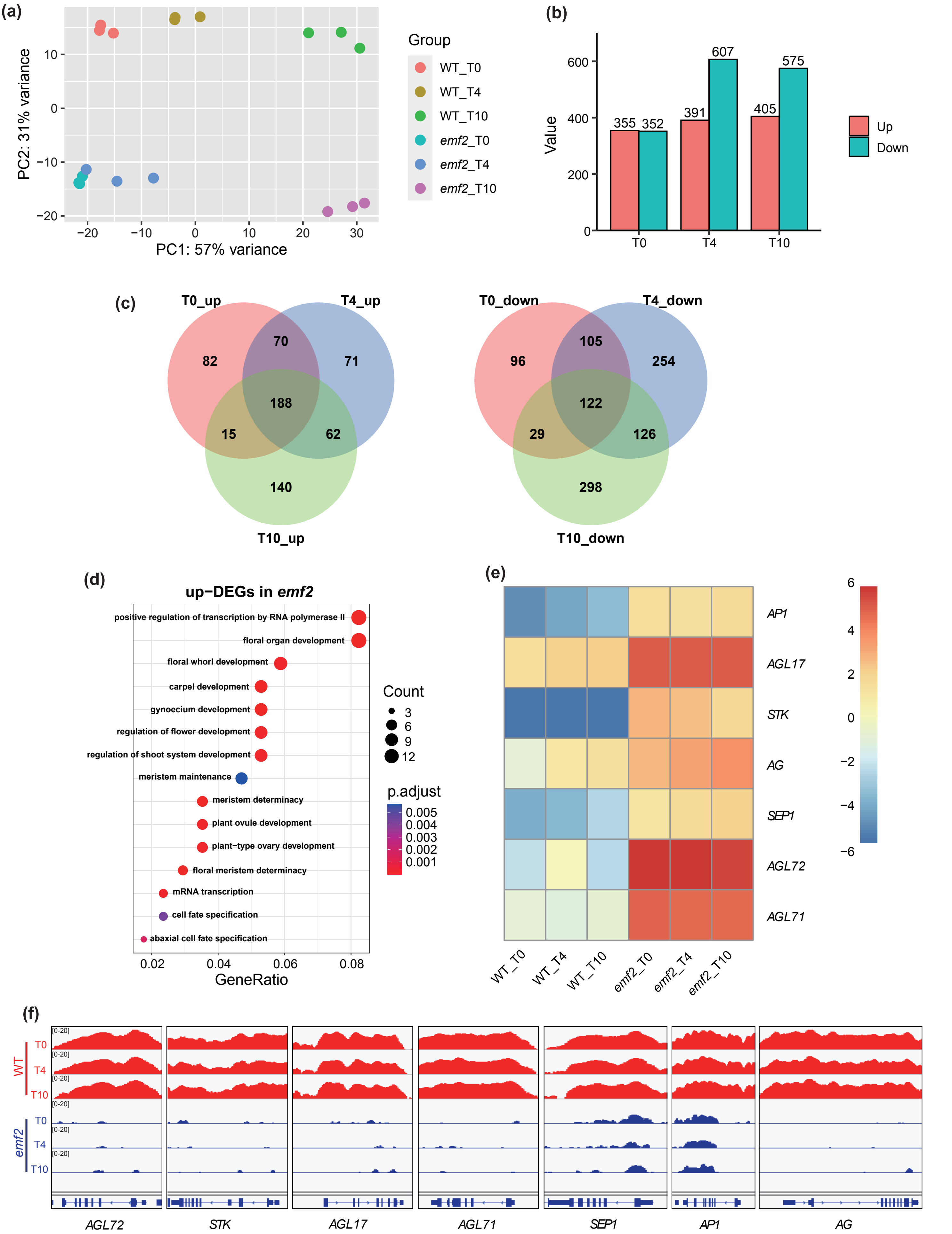

Fig. S1 Overview of transcriptomic profiles between wild-type and *emf2* during root regeneration. (a) Principal component analysis (PCA) of RNA-seq data. (b) Numbers of differentially expressed genes (DEGs) between wild-type and *emf2* (*emf2 v.s.* wild-type) across different time points. (c) Overlap analysis of DEGs in *emf2* during root induction. (d) Gene Ontology (GO) analysis of the consistently up-regulated genes in *emf2*. (e) Expression patterns of MADS-box transcription factor genes in wild-type and *emf2* calli during root induction. The color bar represents the range of log_2(RPKM) values from low (blue) to high (red). (f) IGV snapshots showing H3K27me3 profiles of MADS-box transcription factor gene loci in wild-type and *emf2* calli during root induction.

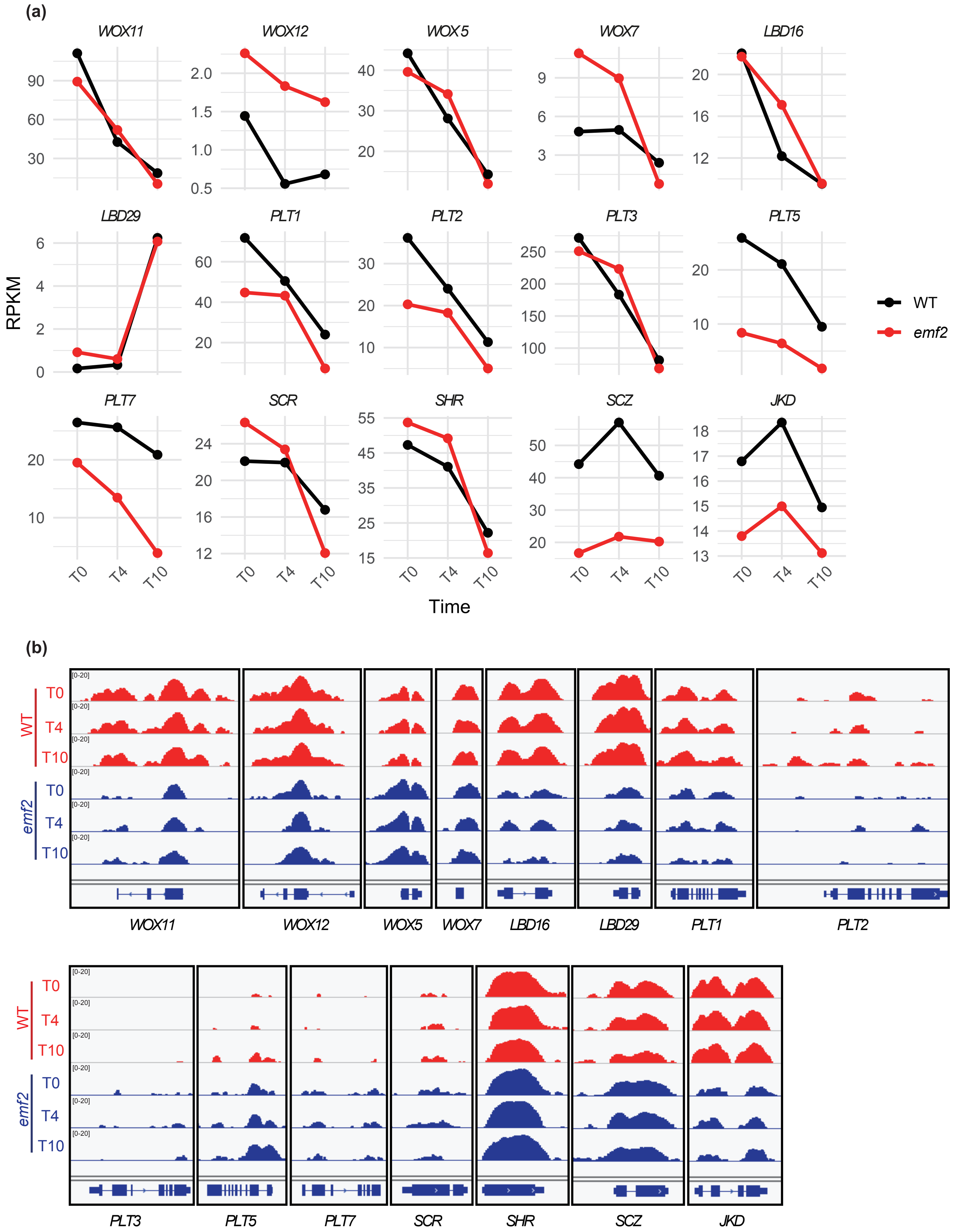

Fig. S2 Patterns of *de novo* root regeneration (DNRR) marker genes in wild-type and *emf2* during root induction. Expression (a) and H3K27me3 profiles (b) of DNRR marker genes in wild-type and *emf2* during root induction. Dots in (a) represent the mean RPKM values from three replicates.

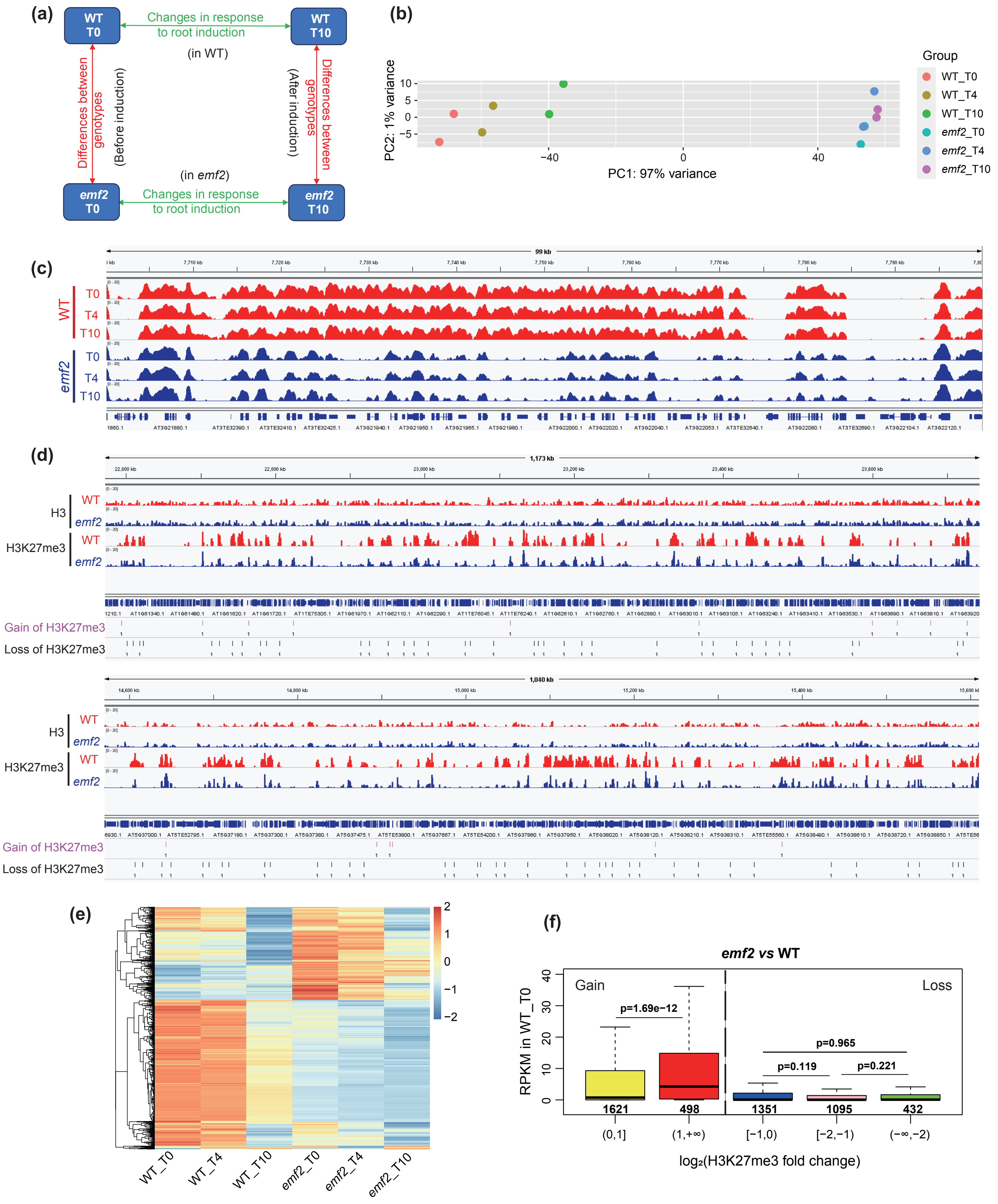

(a)
WT T0
WT T10
Changes in response to root induction
(in WT)
Differences between genotypes
(Before induction)
(After induction)
(in emf2)
emf2 T0
emf2 T10
(b)
PC2: 1% variance
PC1: 97% variance
Group
WT_T0
WT_T4
WT_T10
emf2_T0
emf2_T4
emf2_T10
(c)
99 kb
WT
T0
T4
T10
emf2
(d)
1,173 kb
H3
H3K27me3
Gain of H3K27me3
Loss of H3K27me3
1,040 kb
(e)
WT_T0
WT_T4
WT_T10
emf2_T0
emf2_T4
emf2_T10
(f)
emf2 vs WT
Gain
Loss
RPKM in WT_T0
p=1.69e−12
p=0.965
p=0.119
p=0.221
1621
498
1351
1095
432
(0,1]
(1,+∞)
[−1,0)
[−2,−1)
(−∞,−2)
log2(H3K27me3 fold change)

(g)

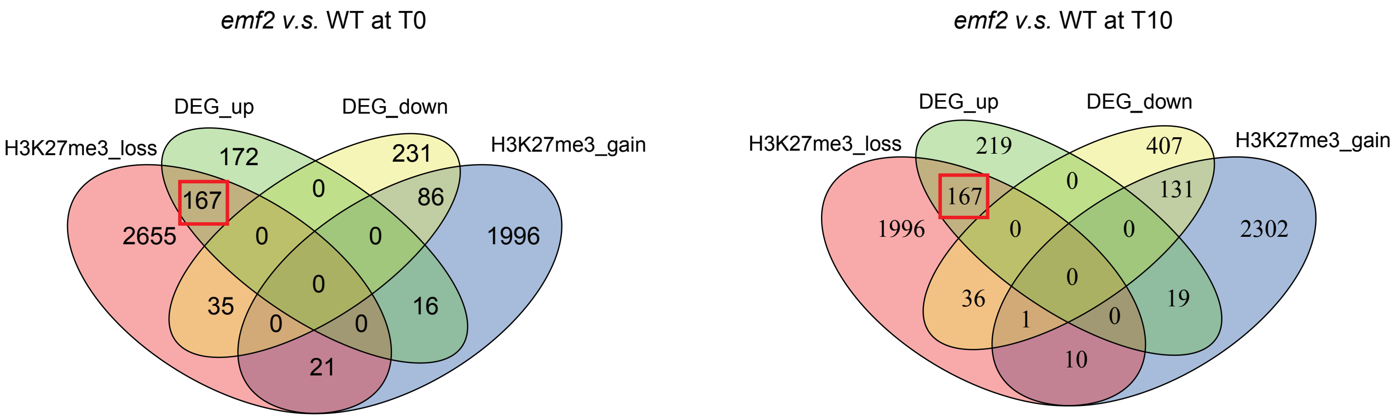

(h)

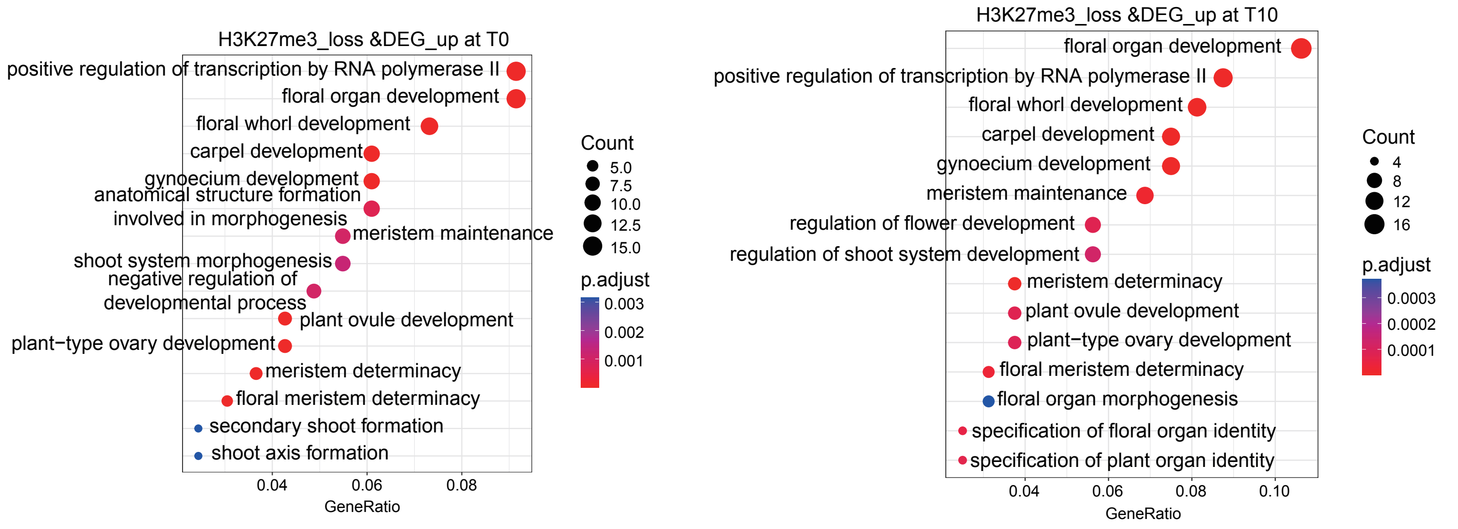

Fig. S3 Comparison of H3K27me3 differentially enriched sites within and between genotypes during root regeneration. (a) Strategies used to identify differentially enriched sites (DESs) between samples. (b) Principal component analysis (PCA) of ChIP-seq data. (c) IGV snapshot showing H3K27me3 levels in a 100 kb region across different samples. (d) IGV snapshots showing the comparison of H3 and H3K27me3 levels between wild-type and *emf2* at T0. Differentially enriched sites (DESs) of H3K27me3 in *emf2* are displayed in individual tracks, with pink indicating gain of H3K27me3 and black indicating loss of H3K27me3 relative to wild-type. (e) Dynamics of H3K27me3 for genes located at sites that are enriched for H3K27me3 in wild-type at T0 but show reduced levels at T10. H3K27me3 enrichment was calculated as the mean H3K27me3/H3 signal across gene bodies. For comparison across samples, enrichment values were row-scaled (mean-centered and variance-standardized), with the color scale indicating relative H3K27me3 levels from low (blue) to high (red). (f) Expression levels of genes located within DESs in the wild-type background. The box plots indicate the median (line within the box), the lower and upper quartiles (box), margined by the largest and smallest data points that are still within the interval of 1.5 times the interquartile range from the box (whiskers). *p* values indicate the two-sided Mann-Whitney U-test results. (g) Overlap analysis of genes exhibiting differential H3K27me3 enrichment and differential expression in *emf2* at T0 and T10. Genes showing both loss of H3K27me3 and upregulation in *emf2* are highlighted with red rectangles. (h) Gene Ontology (GO) analysis of genes showing both loss of H3K27me3 and upregulation in *emf2* at T0 and T10.

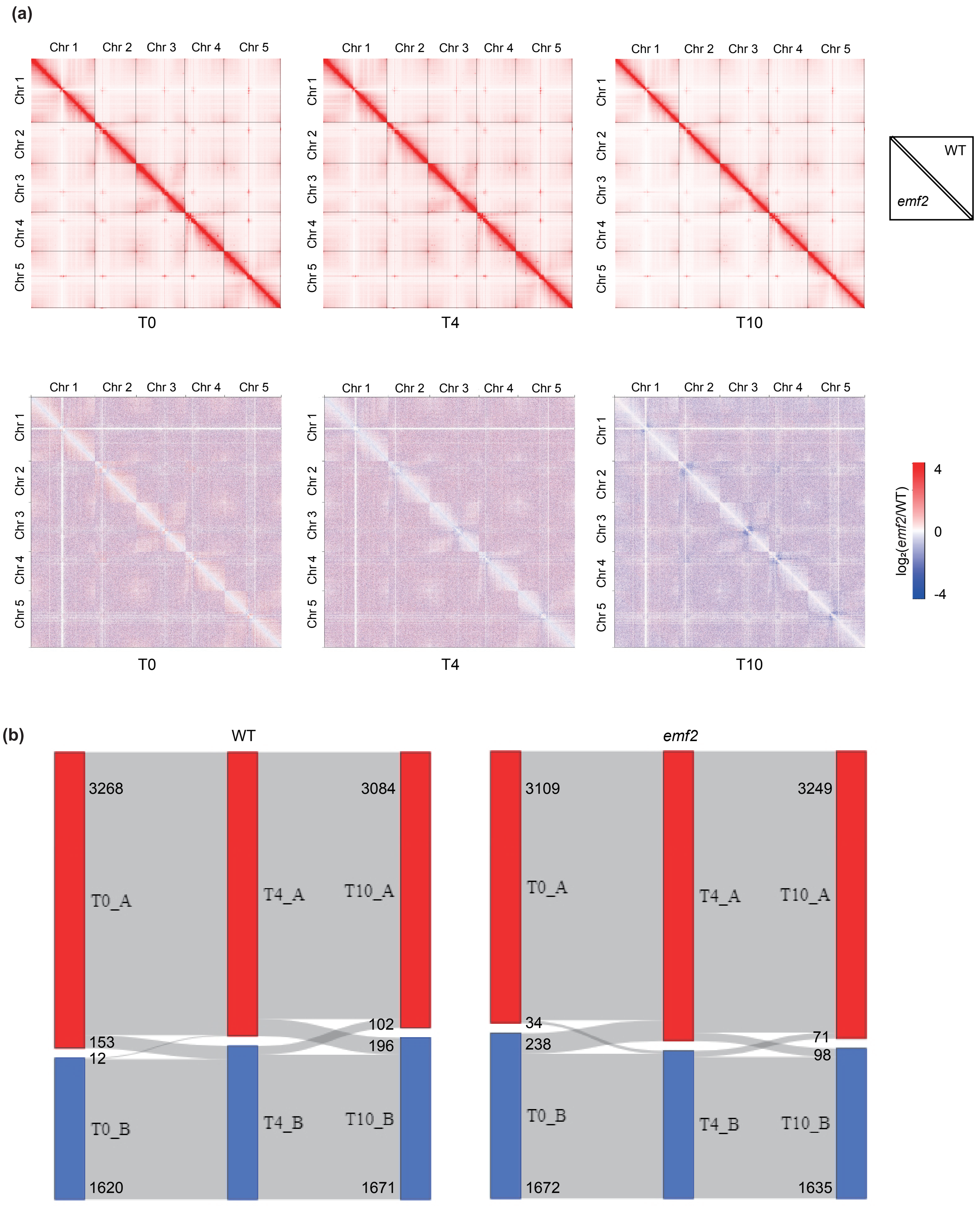

Fig. S4 Genome-wide chromatin interaction analyses of wild-type and *emf2* across different time points. (a) Chromatin contact maps of wild-type and *emf2* during root induction.Top panels: Comparison of Hi-C contact maps between wild-type and *emf2*. Bottom panels: Differential interaction metrices showing chromatin contact differences between wild-type and *emf2*. Colours for the bottom panels indicate the difference in chromatin interaction strengths from low (blue) to high (red). (b) Sankey diagrams illustrating A/B compartment transitions during root induction in wild-type and *emf2*, respectively. Numbers on the flows indicate the number of 20kb genomic bins in each corresponding category.

(a)

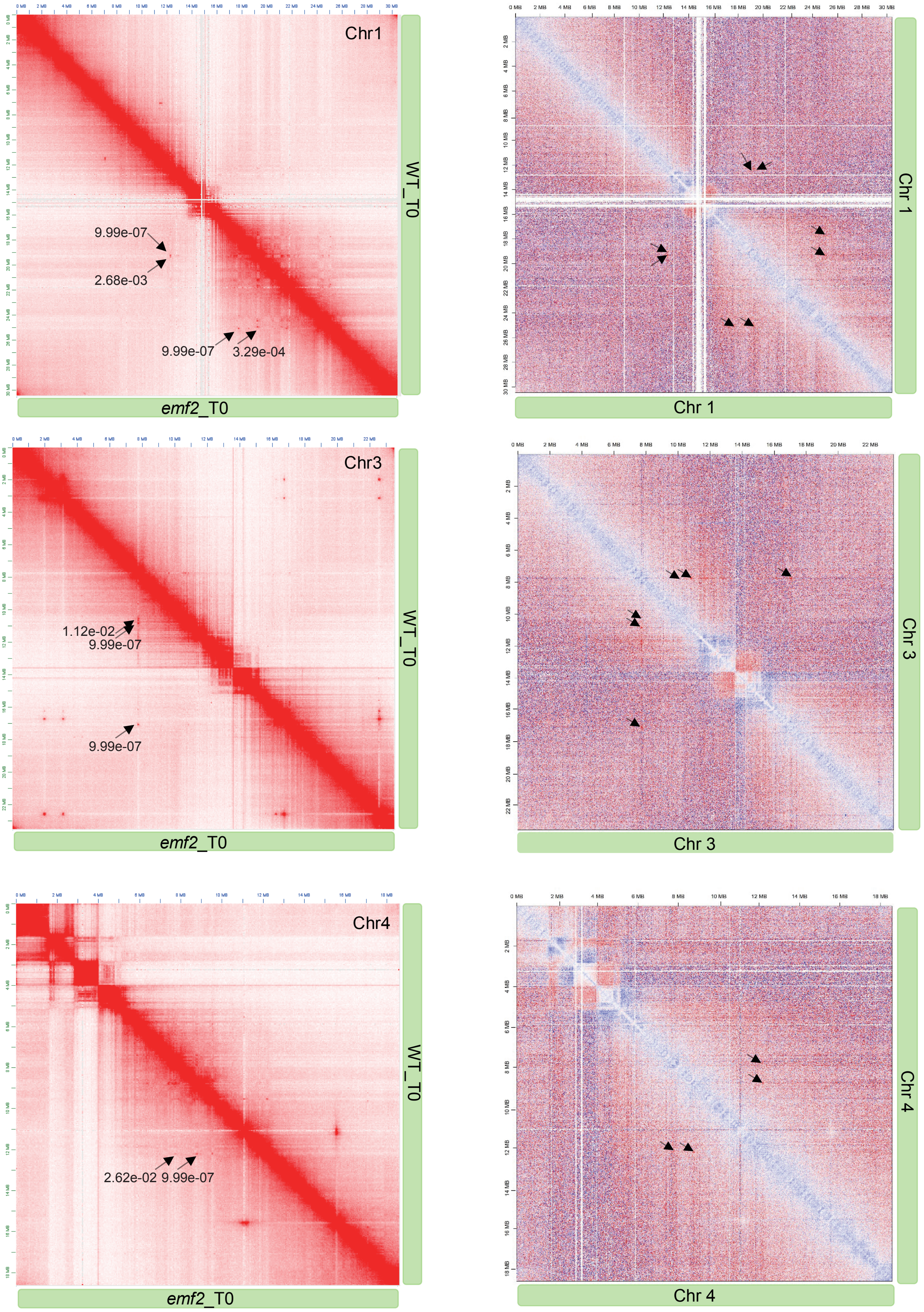
Chr1
WT_T0
9.99e-07
2.68e-03
9.99e-07
3.29e-04
emf2_T0
Chr 1
Chr 1
Chr3
WT_T0
1.12e-02
9.99e-07
9.99e-07
emf2_T0
Chr 3
Chr 3
Chr4
WT_T0
2.62e-02
9.99e-07
emf2_T0
Chr 4
Chr 4

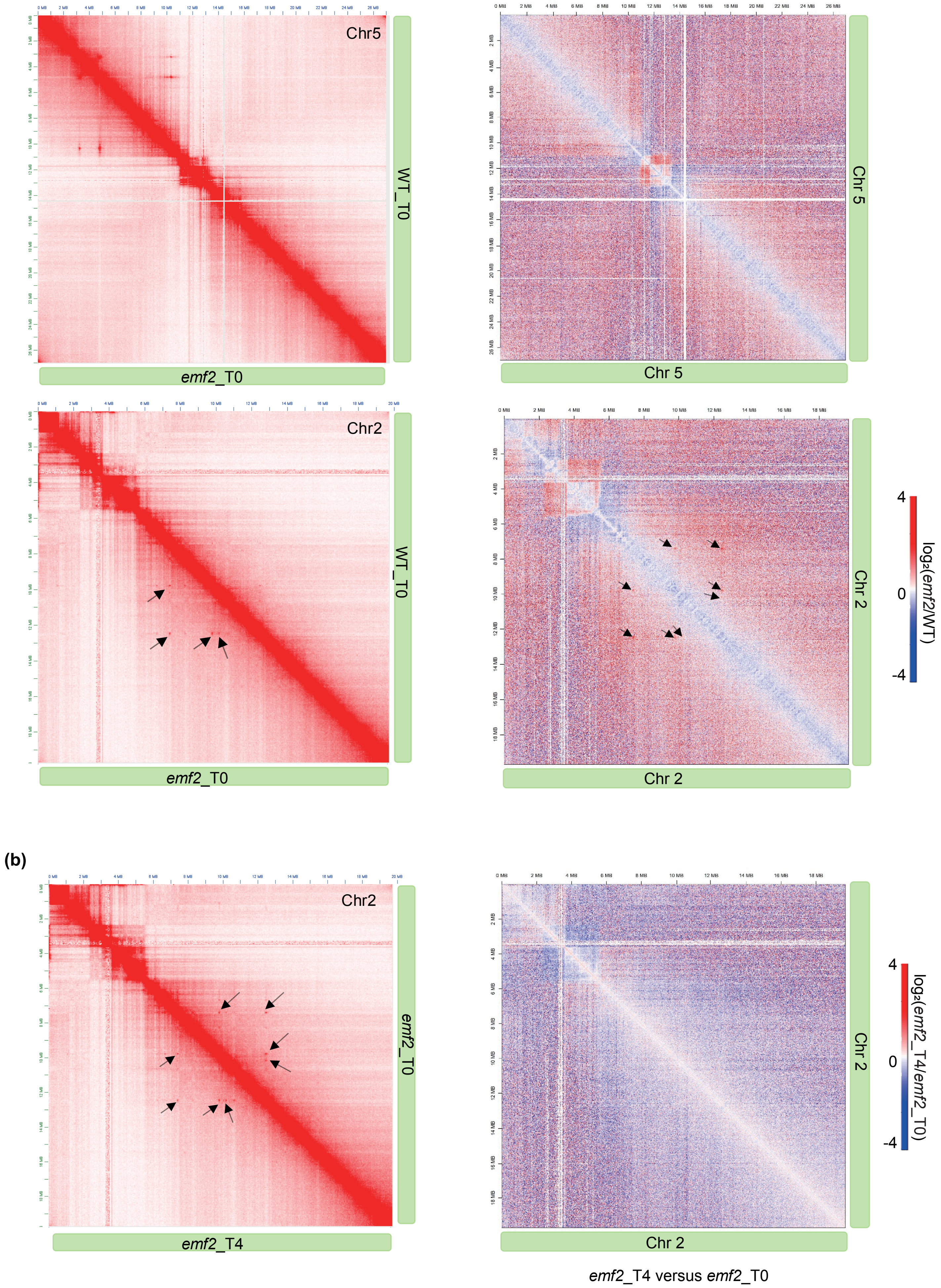

Chr5
WT_T0
emf2_T0
Chr 5
Chr2
WT_T0
emf2_T0
Chr 2
4
0
-4
log2(emf2/WT)
(b)
Chr2
emf2_T0
emf2_T4
Chr 2
4
0
-4
log2(emf2_T4/emf2_T0)
emf2_T4 versus emf2_T0

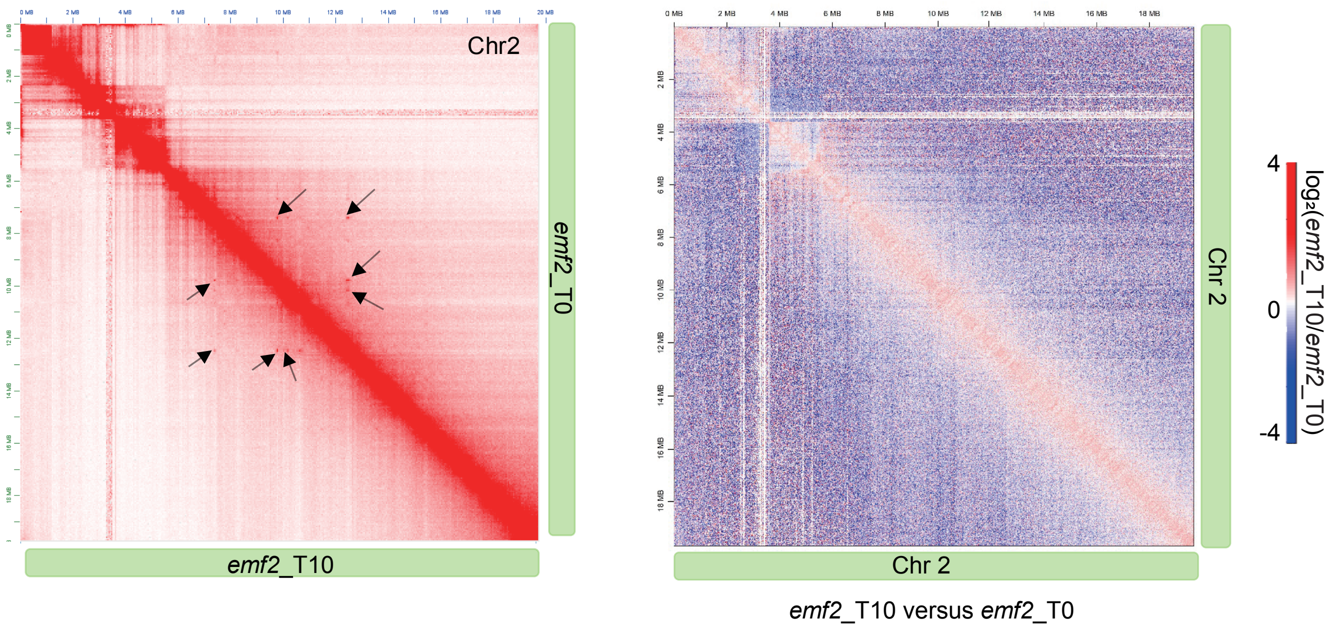

Fig. S5 Comparisons of Hi-C maps between wild-type and *emf2* during root induction. (a) Left panels: Chromosome-scale Hi-C maps comparing wild-type and *emf2*. The chromosome 2 map shown here is also presented in Fig 3a. Right panels: Differential interaction maps comparing *emf2* and wild-type at T0. (b) Left panels: Hi-C map comparisons of chromosome 2 in *emf2* across different time points during root induction. Right panels: Differential interaction maps comparing chromatin contact strengths across different time points during root induction in *emf2* on chromosome 2. Arrows in (a) and (b) highlight the *emf2*-specific long-range chromatin interactions, and the numbers adjacent to the arrows in (a) represent empirical *p* values based on 10^6 simulations comparing contacts between wild-type and *emf2*. Colours in the right panels indicate differences in chromatin interaction strength, ranging from low (blue) to high (red).

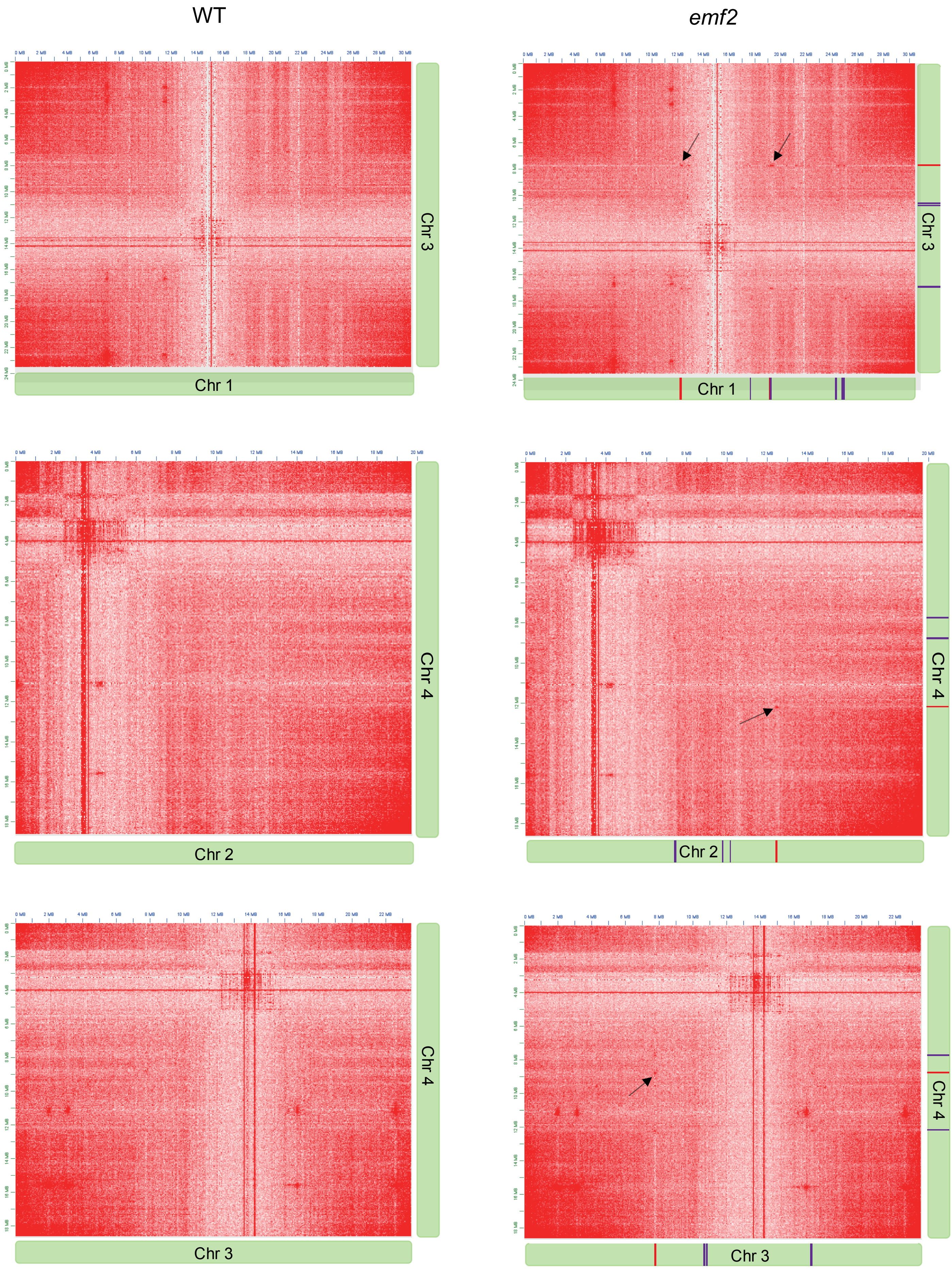

Fig. S6 Hub regions involved in new long-range chromatin interactions in *cis* form *trans* chromatin interactions in *emf2*. Arrows highlight the *trans* chromatin interactions in *emf2*. Strips indicate the locations of interaction regions identified in *emf2*, with the red ones represente regions forming *trans* chromatin interactions in *emf2*.

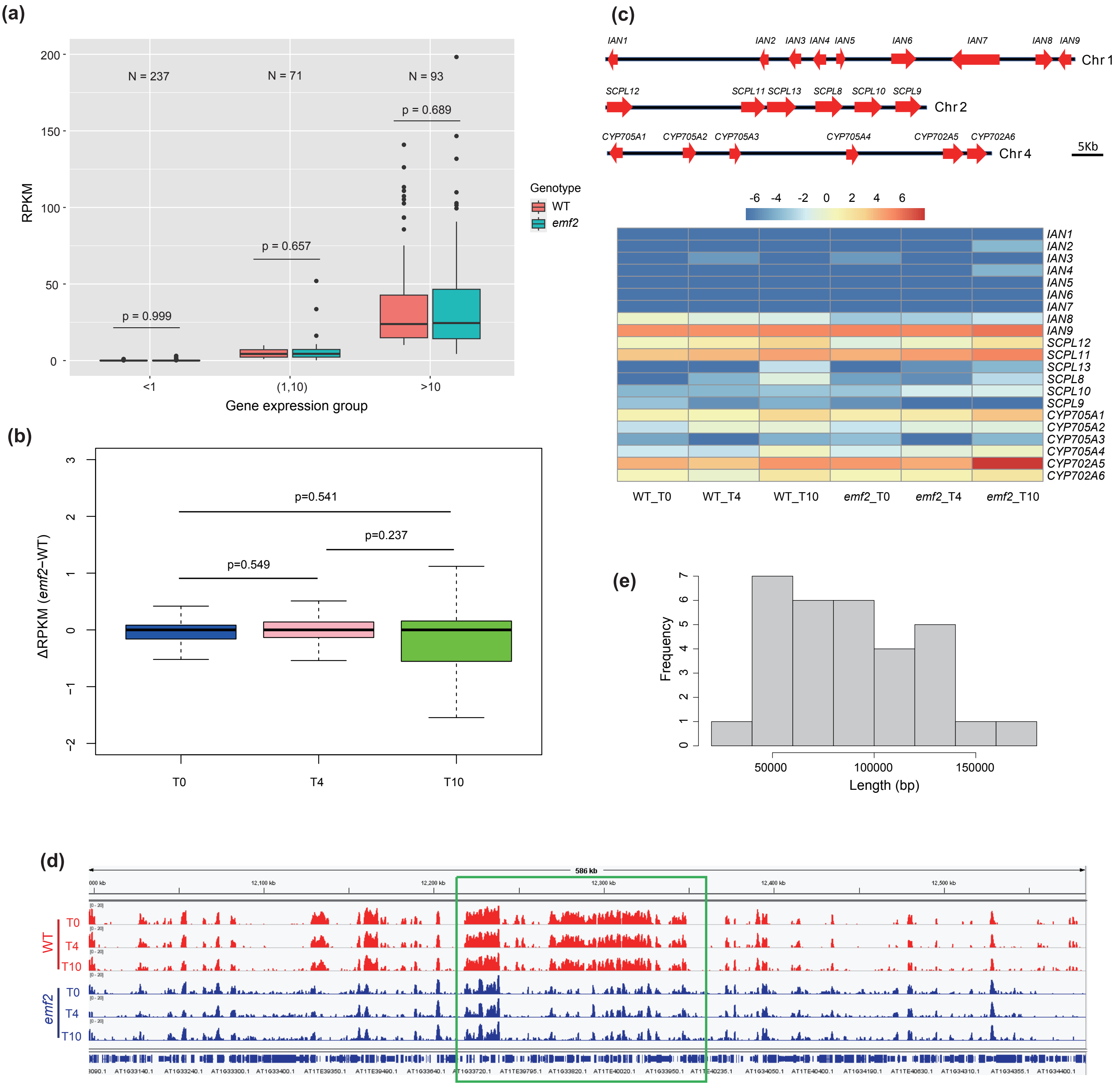

Fig. S7 Characteristics of the *emf2*-specific long-range interaction regions. (a) Genes residing in *emf2*-specific interaction regions show comparable expression levels between wild-type and *emf2* at T0. Genes in these regions were categrozied into three groups based on their expression levels in wild-type. (b) Expression comparison of genes located within *emf2*-specific interaction regions between wild-type and *emf2* at different time points. (c) Top panel: Identification of gene clusters within the *emf2*-specific interaction regions. Bottom panel: Expression of clustered genes in wild-type and *emf2* calli during root induction. The color scale represents the range of $\log_2$(RPKM+0.01) values from low (blue) to high (red). (d) IGV snapshot comparing H3K27me3 levels between *emf2*-specific interaction regions and adjacent regions across different samples. The green rectangle highlights one of the *emf2*-specific interaction regions. (e) Size distribution of the *emf2*-specific interaction regions. The box plots in (a) and (b) indicate the median (line within the box), the lower and upper quartiles (box), margined by the largest and smallest data points that are still within the interval of 1.5 times the interquartile range from the box (whiskers). *p* values indicate the two-sided Mann-Whitney U-test results.

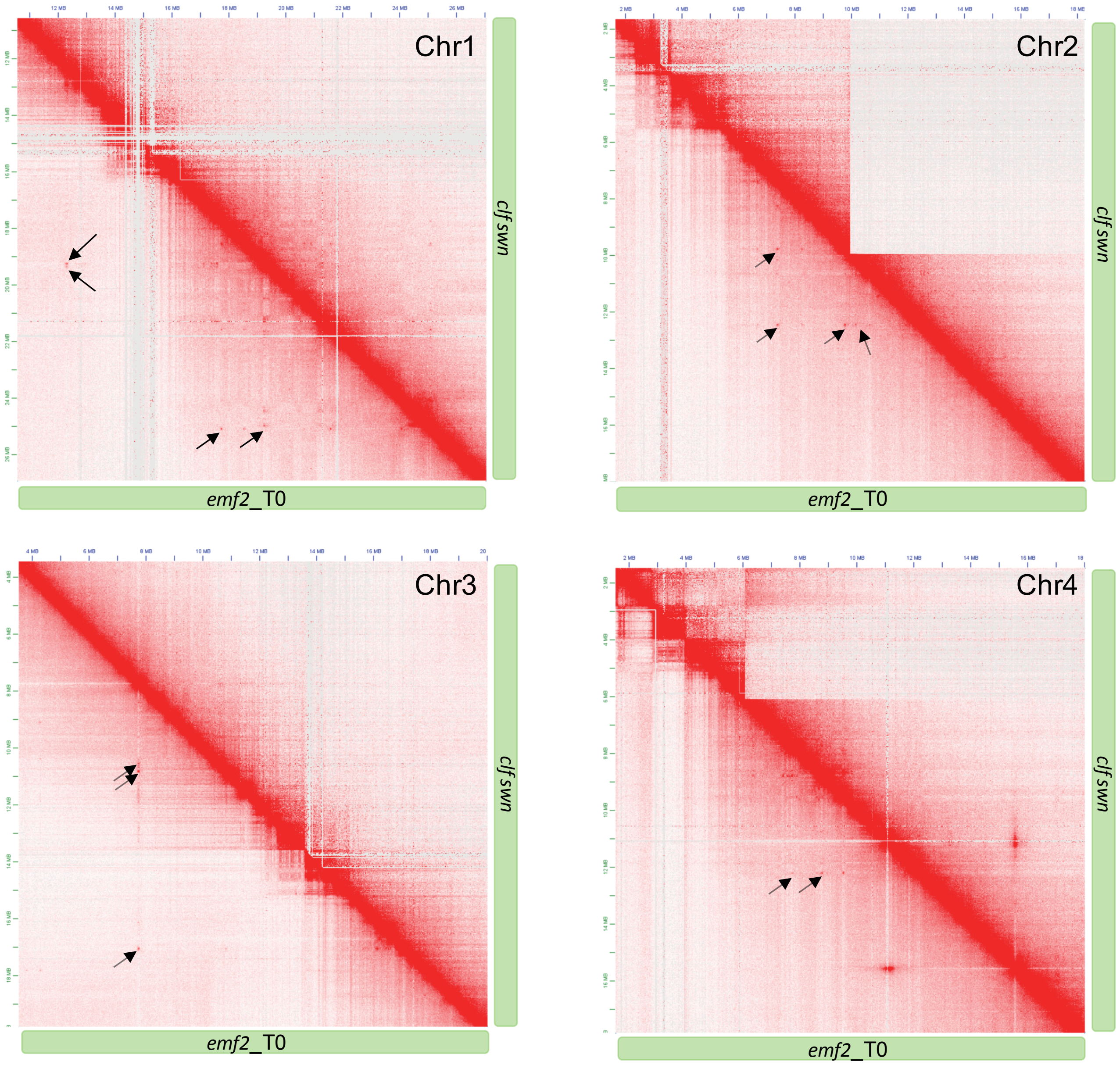

Fig. S8 Comparison of Hi-C maps between *emf2* and *clf swn*. Hi-C data for *clf swn* were obtained from a published study (Feng *et al*., 2014). Arrows highlight the new long-range chromatin interactions detected in *emf2*. A translocation between chromosome 2 and 4 is present in *clf swn*.

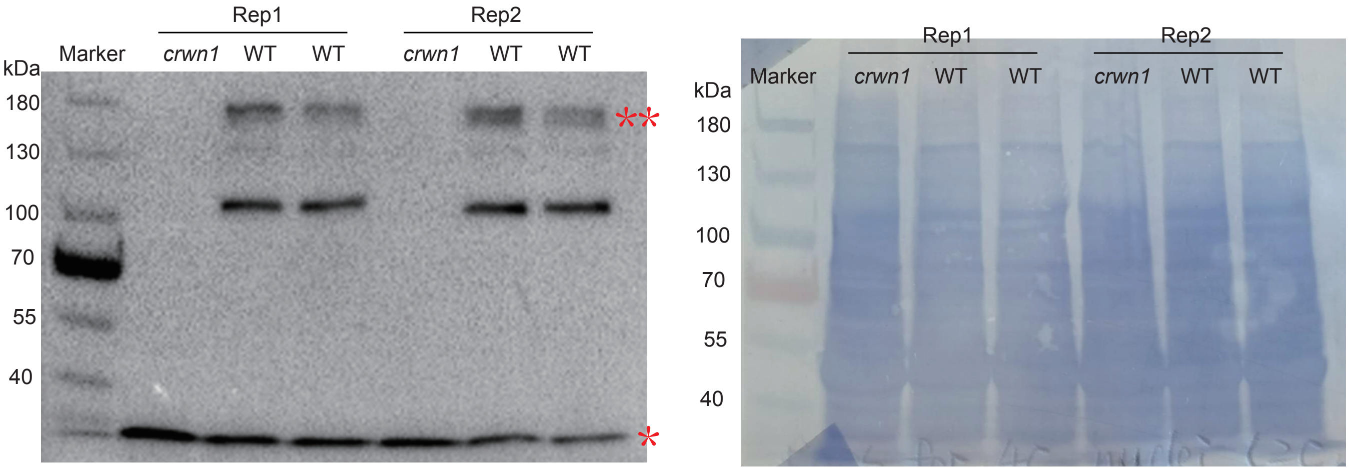

Fig. S9 Detection of CRWN1 using an endogenous antibody by Western blot. The left panel shows immunoblot signals, while the right panel presents the loading control visualized by Coomassie Blue staining. The asterisk (*) denotes a non-specific band, and the double asterisks (**) indicate a potential band corresponding to the CRWN1 dimer. Rep1 and Rep2 represent two biological replicates.

(a)

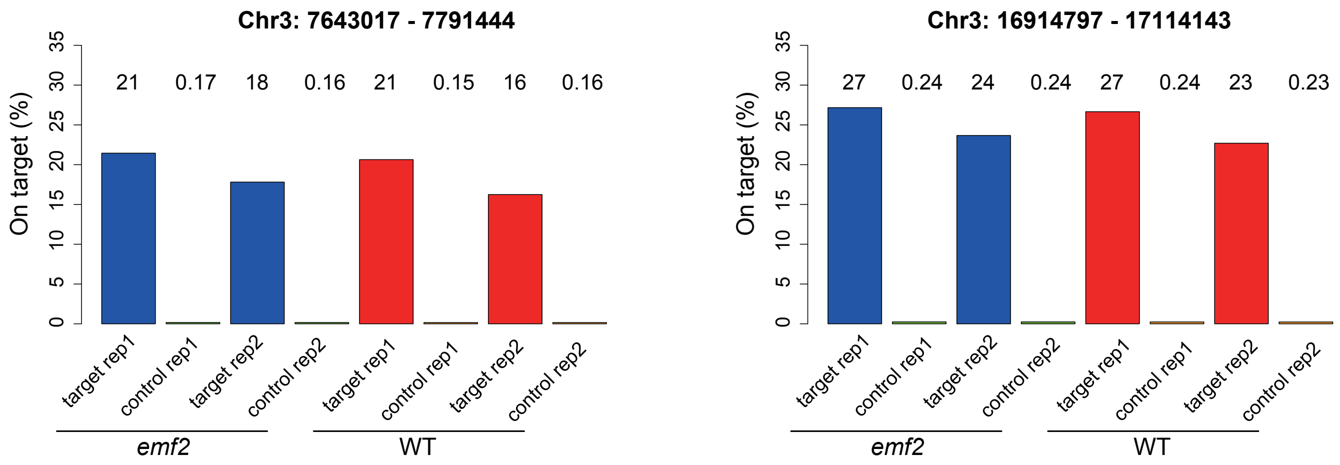

(b)

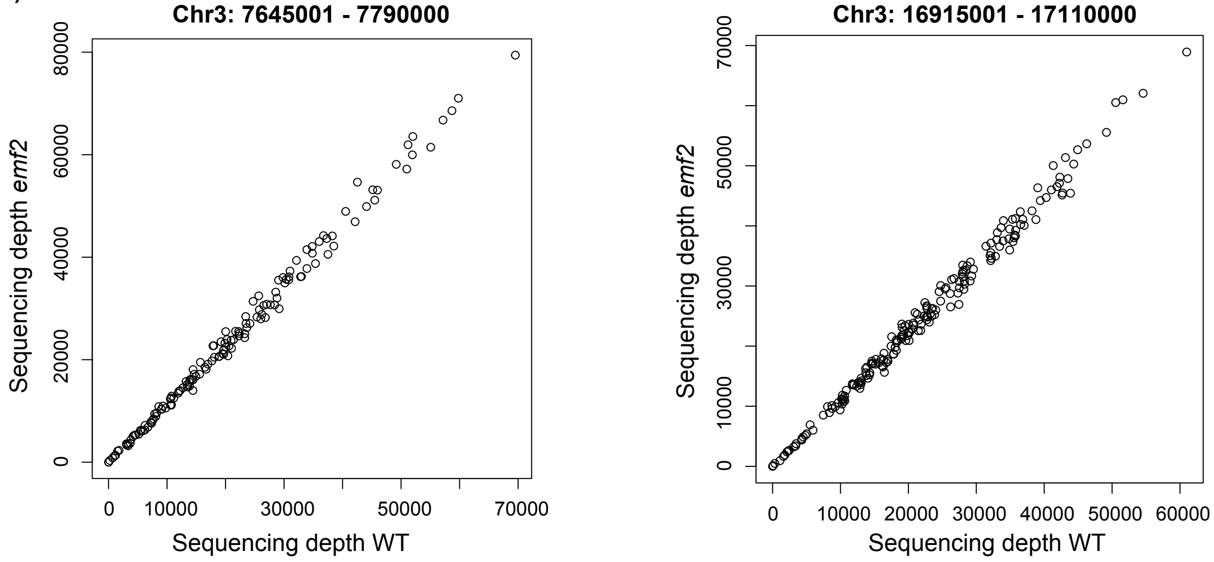

Fig. S10 Enrichment of target regions with BAC-based Capture Hi-C. (a) Fraction of on-target Hi-C reads, defined as those reads overlapping with genomic regions covered by probes, before and after BAC-based Capture Hi-C. (b) Sequencing depths (bin size: 1kb) of the target regions in wild-type and *emf2* BAC-based Capture Hi-C libraries.

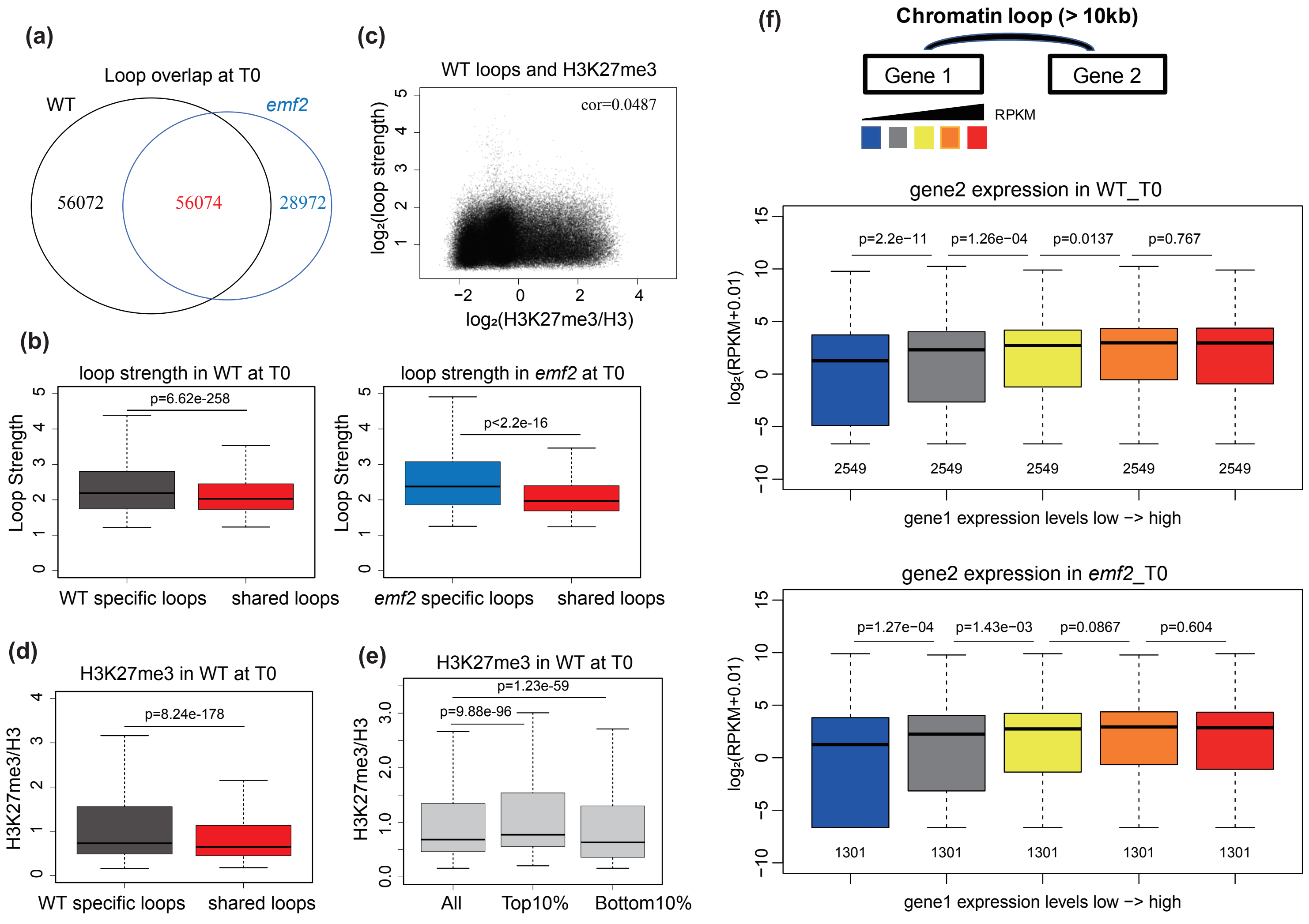

Fig. S11 Relationship between loop strength, H3K27me3, and gene expression at T0. (a) Overlap analysis of chromatin loops identified in wild-type and *emf2* at T0. (b) Comparison of loop strength between loops shared by both genotypes and genotype-specific loops at T0. (c) Correlation between loop strength and H3K27me3 levels in wild-type at T0. The correlation coefficient was calculated using Pearson's correlation test. (d) Comparison of H3K27me3 levels between loops shared by both genotypes and wild-type-specific loops at T0. (e) H3K27me3 levels in loops ranked in the top and bottom 10% by loop strength. (f) Gene interactions *in cis* are associated with gene expression. Gene pairs forming loops longer than 10 kb were grouped into five categories based on the expression level of "gene 1", and the expression levels of "gene 2" were examined in the corresponding genetic background. The box plots in (b) and (d-f) indicate the median (line within the box), the lower and upper quartiles (box), margined by the largest and smallest data points that are still within the interval of 1.5 times the interquartile range from the box (whiskers). p values indicate the two-sided Mann-Whitney U-test results.

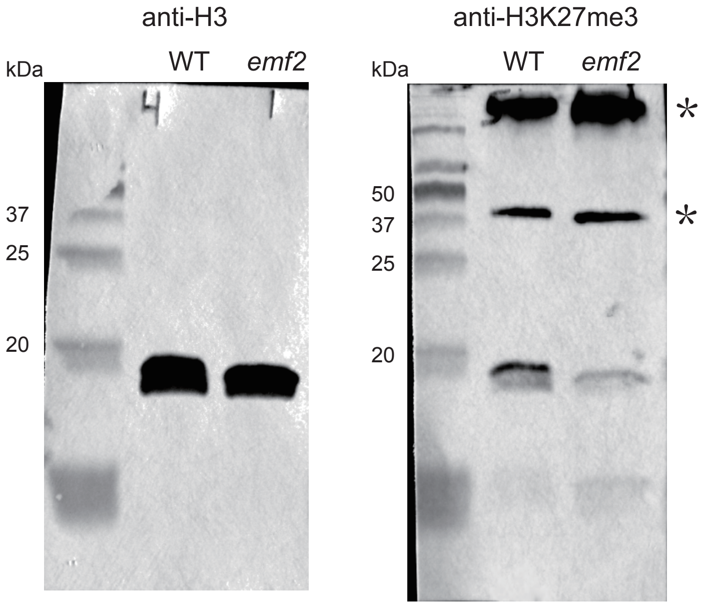

Fig. S12 Uncropped Western blot images related to Fig. 2C. Asterisks (*) indicate non-specific bands.

Table S1 Information of bacterial artificial chromosomes (BACs) used in this work

BAC name	Chromosome	From	To	Length	Signal color
F12M12	3	16914797	17021527	106730	green
F18L15	3	17013815	17114143	100328	
MSD21	3	7643017	7702938	59921	red
MEK6	3	7702630	7709438	6808	
MZN24	3	7709096	7791444	82348	

Table S2 DEGs between *emf2* and wild-type

Table S3 List of cluster genes during root induction

Table S4 Information of DESs identified between samples

Table S5 List of long-range interaction regions and genes located within these regions

Table S6 Information of chromatin loops identified in wild-type and *emf2*

Table S7 Information of gene pairs identified in wild-type and *emf2* at T0

(These Tables are uploaded as separate excel file.)

Reference

Feng S, Cokus SJ, Schubert V, Zhai J, Pellegrini M, Jacobsen SE. 2014. Genome-wide Hi-C analyses in wild-type and mutants reveal high-resolution chromatin interactions in Arabidopsis. *Molecular cell* **55**: 694–707.
